# The Mitochondrial LSU rRNA Group II Intron of *Ustilago maydis* Encodes an Active Homing Endonuclease Likely Involved in Intron Mobility

**DOI:** 10.1371/journal.pone.0049551

**Published:** 2012-11-14

**Authors:** Anja Pfeifer, Bettina Martin, Jörg Kämper, Christoph W. Basse

**Affiliations:** Department of Genetics, Institute for Applied Biosciences of the Karlsruhe Institute of Technology (KIT), Karlsruhe, Germany; University of Massachusetts Medical School, United States of America

## Abstract

**Background:**

The *a2* mating type locus gene *lga2* is critical for uniparental mitochondrial DNA inheritance during sexual development of *Ustilago maydis*. Specifically, the absence of *lga2* results in biparental inheritance, along with efficient transfer of intronic regions in the large subunit rRNA gene between parental molecules. However, the underlying role of the predicted LAGLIDADG homing endonuclease gene I-*Uma*I located within the group II intron LRII1 has remained unresolved.

**Methodology/Principal Findings:**

We have investigated the enzymatic activity of I-*Uma*I *in vitro* based on expression of a tagged full-length and a naturally occurring mutant derivative, which harbors only the N-terminal LAGLIDADG domain. This confirmed Mg^2+^-dependent endonuclease activity and cleavage at the LRII1 insertion site to generate four base pair extensions with 3′ overhangs. Specifically, I-*Uma*I recognizes an asymmetric DNA sequence with a minimum length of 14 base pairs (5′-GACGGGAAGACCCT-3′) and tolerates subtle base pair substitutions within the homing site. Enzymatic analysis of the mutant variant indicated a correlation between the activity *in vitro* and intron homing. Bioinformatic analyses revealed that putatively functional or former functional I-*Uma*I homologs are confined to a few members within the Ustilaginales and Agaricales, including the phylogenetically distant species *Lentinula edodes*, and are linked to group II introns inserted into homologous positions in the LSU rDNA.

**Conclusions/Significance:**

The present data provide strong evidence that intron homing efficiently operates under conditions of biparental inheritance in *U. maydis*. Conversely, uniparental inheritance may be critical to restrict the transmission of mobile introns. Bioinformatic analyses suggest that I-*Uma*I-associated introns have been acquired independently in distant taxa and are more widespread than anticipated from available genomic data.

## Introduction

Homing endonuclease genes (HEGs) are widespread in microbial genomes. They frequently exist in self-splicing group I and group II introns, but also in archaeal introns, intein coding sequences and phage genomes [1]–[3]. On the basis of conserved amino acid motifs, at least five families of homing endonuclease (HE) proteins are distinguished [3]–[7]. The largest known family, termed the LAGLIDADG homing endonucleases (LHEs), is primarily encoded within archaea and in the mitochondrial or chloroplast genomes of algae and fungi [5], [6]. LAGLIDADG enzymes contain one or two copies of the consensus motif. Specifically, single-motif enzymes function as homodimers, whereas double-motif enzymes are monomers with two separate domains, each resembling a subunit of a single LAGLIDADG protein [5]. LAGLIDADG motifs are not only restricted to homing endonucleases, but also exists in other proteins, such as the HO endonuclease, which in yeast mediates the mating type switch [8], [9].

HEG-containing introns are generally considered opportunistic selfish elements, with the ability to spread within or between genomes if the corresponding homing sites are present on recipient DNA [10]. Cleavage by homing endonucleases differs from restriction enzymes in that longer target sites, with lengths between 14 to 40 base pairs, are recognized [1], [3], [5], [11]. These sites match exactly the intron insertion site in donor DNA, meaning that only DNA is cut that does not contain a copy of the intron interrupting the target site. HEG-containing introns are mobilized by gene conversion, which is initiated by double strand cleavage within the intronless allelic sites on recipient DNA. Subsequent recombination using the intron-containing copy as template confers a homozygous state with two intron-containing alleles, while the intronless allele is lost [2], [3], [5], [11], [12]. Intron homing occurs with efficiencies close to 100% as exemplified from transfer of the I-*Sce*I-containing mitochondrial group I intron omega (ω) in combinations of *Saccharomyces cerevisiae* ω^+^ and ω^−^ cells [5], [13], [14], [15]. However, in the majority of sexual eukaryotes, uniparental mitochondrial DNA (mtDNA) inheritance efficiently prevents recombination between parental mtDNAs making it difficult to address an underlying role of HEGs [16], [17].

**Figure 1:**
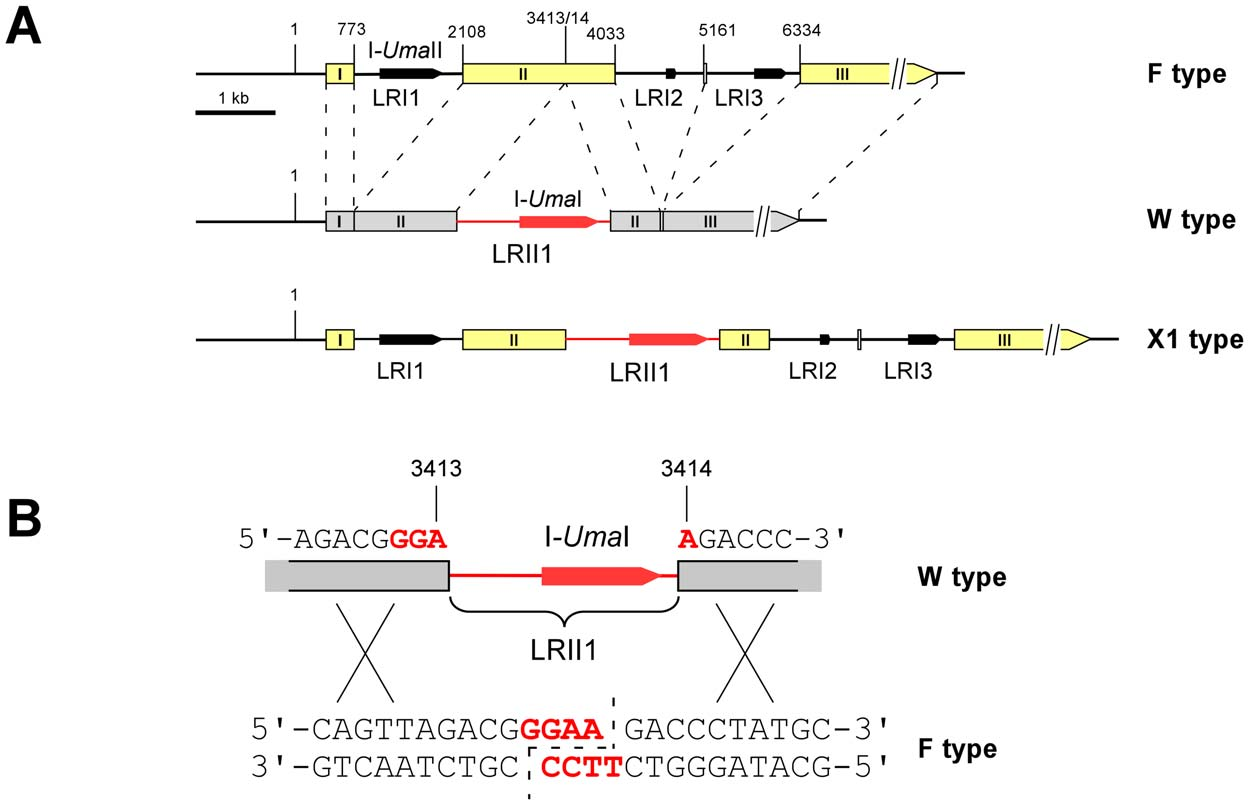
Determination of the I-*Uma*I cleavage site. (A) Schematic of the polymorphic region within the LSU rDNA of *U. maydis*. Depicted are the parental W and F types and the recombinant X1 type. Boxes refer to deduced exon sequences (the three major ones are numbered from I to III). Stippled lines connect homologous sequences. All numbers refer to the complete mitochondrial genome sequence of *U. maydis* strain 521. Black arrows mark predicted HEGs within intronic regions (termed LRI1, LRI2, LRI3 and LRII1). LRII1 and I-*Uma*I are marked in red. The schematic (drawn to scale) is adapted from Fedler *et al.* [18] with permission of the Genetics Society of America. (B) The schematic shows the target cleavage site (red, bold face type) within the recipient F type sequence (indicated positions refers to the mitochondrial genome sequence of strain 521; see part A). The staggered line (stippled) marks produced 3′ overhangs. The upper part shows the donor sequence of the W type interrupted by intron LRII1. Crosses refer to potential homologous recombination events.

Previously, we identified a restriction length polymorphisms in the mitochondrial large subunit (LSU) ribosomal RNA (rRNA) gene of the maize smut fungus *Ustilago maydis* [18]. Within this polymorphic region, the individual mitochondrial genotypes (mitotypes) differ in the number and position of HEG-containing group I and group II introns. In particular, the W type differs from the F type by the presence of the group II intron LRII1, but lacks flanking F type-associated group I introns (Figure 1A). Investigation of uniparental mtDNA inheritance revealed that mtDNA was preferentially transmitted from the *a2* partner by virtue of the *a2* mating type locus genes *lga2* and *rga2*. Hereby, *lga2* mediates loss of *a1*-associated mtDNA, while *rga2* protects *a2*-associated mtDNA from *lga2*-mediated elimination. This provided for conditions of biparental inheritance established either in the absence of *lga2* or in the presence of an *a1* partner ectopically expressing *rga2* [18]. Interestingly, under these conditions, recombinant mtDNA molecules were efficiently produced, and apparently, this proceeded in an unidirectional manner. Specifically, in combinations of F and W types, the F type was almost completely lost in favor of the recombinant X1 type, which matched the parental F type, but additionally carried the W type-derived LRII1 intron (Figure 1A; [18]). This intron contains a predicted HEG (here termed I-*Uma*I according to the corresponding nomenclature convention; [19]), raising the question of underlying intron homing. In addition, we previously identified a W type strain (MF18) in which due to a naturally occurring frameshift mutation the second LAGLIDADG domain of I-*Uma*I is not expressed. Only in combinations with this strain, the parental F type was maintained and significantly less recombinant X1 type was produced [18]. This suggested a role of I-*Uma*I in the mobilization of the LRII1 intron.

To provide firm evidence for intron homing among mitochondrial LSU rRNA genes, we have analyzed enzymatic activities of I-*Uma*I and a mutated variant lacking the second LAGLIDADG motif. In addition, we have determined the target site specificity. Here, we show that I-*Uma*I represents a Mg^2+^-dependent endonuclease requiring both LAGLIDADG domains for activity and recognizing a minimum target site of 14 base pairs, which defines the LRII1 insertion site. The gained insight has further been exploited to make predictions on the existence of putatively functional I-*Uma*I homologs sharing the same cleavage specificity.

## Results

### Expression of I-*Uma*I

**Figure 2:**
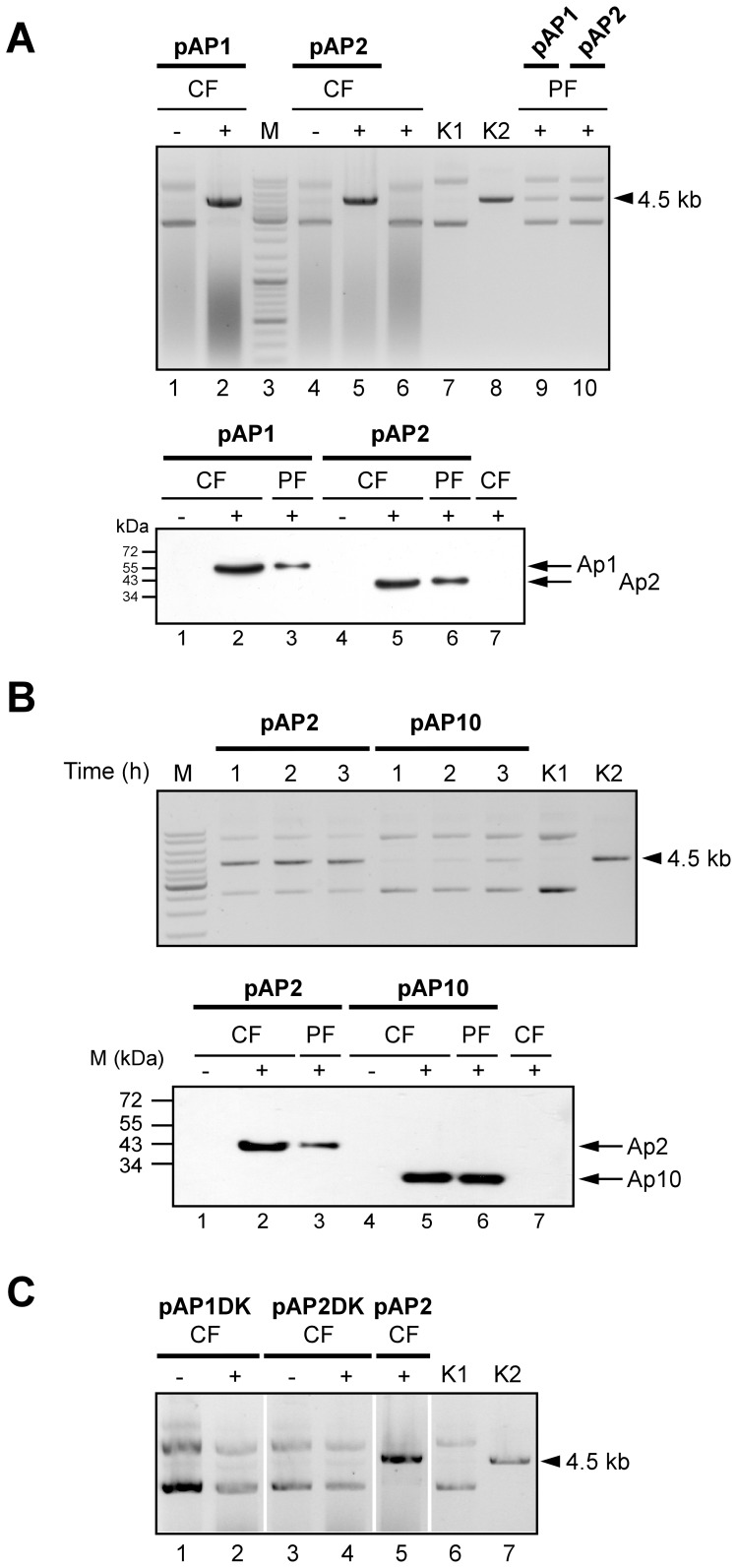
Enzymatic analysis of I-*Uma*I in dependence on the two LAGLIDADG domains. (A) Cleavage analysis with crude fractions (CF) from either non-induced (−) or induced (+) pAP1 (1,2) and pAP2 (4,5) *E. coli* cells in the presence of substrate plasmid pSL521. CF from non-transformed *E. coli* cells incubated under inducing conditions served as a negative control (6). Cleavage analysis with purified fractions (PF) from induced pAP1 (9) and pAP2 (10) cells. DNA ladder: thick bands correspond to 500, 1000 and 3000 bp (3). The bands directly above 3 kb are 3.5, 4 and 5 kb. pSL521 uncut (K1) or linearized (K2) served as markers. The size of pSL521 is 4503 bp (arrowhead). Bottom panel: verification of I-*Uma*I expression. Protein fractions from either non-induced (-) or induced (+) cells were applied to SDS-PAGE for subsequent immunoblot analysis to detect His-tagged I-*Uma*I expressed in pAP1 (1–3; additional THX domain) and pAP2 (4–6) cells. CF from non-transformed *E. coli* cells incubated under inducing conditions served as a negative control (7). All lanes are from the same blot. The predicted molecular masses for AP1 and AP2 are 55.8 and 42.3 kDa, respectively. (B) Comparison between the enzymatic activities of I-*Uma*I (pAP2) and I-*Uma*I_mut_ (pAP10). PF from induced pAP2 or pAP10 cells was incubated with substrate plasmid pSL521 for 1, 2 and 3 h, respectively. The thick marker (M) band corresponds to 3 kb (see part A). Controls K1, K2 as in (A). Bottom panel: verification of I-*Uma*I_mut_ expression. Protein fractions from either non-induced (−) or induced (+) cells were applied to SDS-PAGE for subsequent immunoblot analysis to detect His-tagged I-*Uma*I_mut_ expressed in pAP10 cells (4–6). The corresponding fractions from pAP2 (1–3) and CF from induced, non-transformed *E. coli* served for controls (7). The predicted molecular mass for AP10 is 27.0 kDa. (A, B) Loaded protein amounts were 16–18 µg for CF and 0.23–0.34 µg for PF. (C) Analysis of AP1DK and AP2DK activities. CF from non-induced (−) or induced (+) pAP1DK (1,2) and pAP2DK (3,4) cells were incubated with substrate plasmid pSL521. The reaction with CF from induced pAP2 cells served for control (5). Controls K1, K2 as in (A). All lanes are from the same blot.

To express the I-*Uma*I gene in *Escherichia coli*, the mitochondrial codon usage of *U. maydis* was determined based on its annotated mitochondrial genome sequence (NCBI accession no. DQ157700) comprising 15 genes for proteins of the respiratory chain complex and 11 putative HEGs within introns of the LSU rRNA, *cox1* and *cob* genes (Table S1). Comparative sequence analysis revealed major differences between the mitochondrial codon usage of *U. maydis* and *S. cerevisiae* (Table S2). In conclusion, except for the very rarely occurring triplets TTA/G and AGG, which were absent from the I-*Uma*I sequence, and the complete absence of the AGA and TGA codons, the mitochondrial code of *U. maydis* basically did not deviate from the standard code. In addition, the rarely occurring ATA codon, which occurs at nucleotide position 787 in the I-*Uma*I sequence, likely encodes Ile (Table S2). Therefore, the I-*Uma*I open reading frame (ORF) required no adaptation for expression in *E. coli*. The I-*Uma*I gene was conditionally expressed with a C-terminal His tag extension. The corresponding construct (pAP1) additionally expressed an N-terminal thioredoxin (THX) domain to assure solubility of the product. In addition, a plasmid (pAP2) was used lacking the THX extension to exclude a possible influence on enzymatic activity. Immunoblot analysis confirmed proper expression of both fusion proteins (here termed AP1 and AP2, respectively) as well as their affinity-based purification by Ni^2+^-NTA column chromatography (Figure 2A).

To assess enzymatic activities of AP1 and AP2, *in vitro* assays were conducted based on cleavage of supercoiled double-stranded plasmid DNA (pSL521), which contains the putative target site region (Figure 1B and Materials and Methods). This revealed that AP1 and AP2 from both crude (CF) or affinity-purified (PF) fractions were able to linearize substrate DNA. As expected, cleavage activity was neither detected in protein extracts from non-induced *E. coli* cells (lanes 1,4 in Figure 2A) nor from non-transformed cells incubated under inducing conditions (lane 6 in Figure 2A).

### Determination of the I-*Uma*I Target Site and Cleavage Conditions

To determine the I-*Uma*I target site, cleaved pSL521 reaction products were blunt ended in the presence of T4 DNA polymerase and religated either in the presence or absence of an intervening DNA fragment (see Materials and Methods). Sequence analysis of three independent clones received from cleavage with either AP1 or AP2 revealed a deletion of four nucleotides (5′-GGAA-3′) at the joining site in all cases. This is in accordance with the feature of LHEs to leave four nucleotide 3′overhangs upon cleavage [3]. Furthermore, this showed that cleavage exactly occurred at the LRII1 insertion site (Figure 1B).

With respect to cleavage conditions, the enzyme worked equally well at 30°C and 37°C, and was active throughout a physiological pH range from 5.5 to 8.5, although cleavage was most efficient at pH values of 8 or 8.5 (Figure S1A). In addition, I-*Uma*I cleaved irrespective of whether the substrate plasmid was linearized or supercoiled (Figure S1B). Homing endonucleases efficiently work in the presence of divalent metal ions like Mg^2+^, Mn^2+^ and Co^2+^ [5], [20] and references therein. The absence of Mg^2+^ in the reaction buffer strongly reduced activity of I-*Uma*I and additional application of 5 mM EGTA abolished substrate cleavage, whereas 0.5 mM Mg^2+^ was sufficient for efficient substrate cleavage. Among additional metal ions (Mn^2+^, Zn^2+^, Co^2+^, Ca^2+^, Cu^2+^, Ni^2+^), only Mn^2+^ provided for cleavage albeit less efficiently than Mg^2+^ (Figure S1C and data not shown).

### Both LAGLIDADG Domains are Required for Enzymatic Activity of I-*Uma*I

The I-*Uma*I sequence contains two predicted LAGLIDADG domains from amino acid positions 48 to 149 and 199 to 308, respectively. While the corresponding ORF sequences of four wild-type isolates were identical and predicted a protein of 336 residues, the ORF sequence of strain MF18 predicted a protein with only the first LAGLIDADG domain due to a frameshift mutation (deletion of nucleotide positions 531–537; [18]). To assess enzymatic activity of this mutant form (here termed I-*Uma*I_mut_), plasmid pAP10 was constructed providing for expression of I-*Uma*I_mut_ up to the naturally occurring stop codon at position 610 (U_610_AG). Due to initial uncertainty about the codon usage for ATA, the single A_547_TA codon was substituted to ATG (see Materials and Methods). However, since this codon lies within the frame-shifted region (starting at nucleotide position 532 in I-*Uma*I*_mut_*), it did not affect the native protein chain. Immunoblot analysis confirmed that AP10 was correctly expressed as His-tagged fusion (Figure 2B). Time-course analysis revealed that the activity of the mutant form was strongly diminished compared to the wild-type form as judged from the appearance of a faint distinct cleavage product after longer incubation (Figure 2B).

To corroborate this finding, plasmids pAP1DK and pAP2DK providing for an internal in frame deletion within the coding region of the second LAGLIDADG domain (amino acid positions 214–249) were constructed. Consistent with a requirement for both LAGLIDADG domains, extracts from corresponding *E. coli* strains lacked homing endonuclease activity (Figure 2C).

### Determination of the I-*Uma*I Target Site Specificity

**Figure 3:**
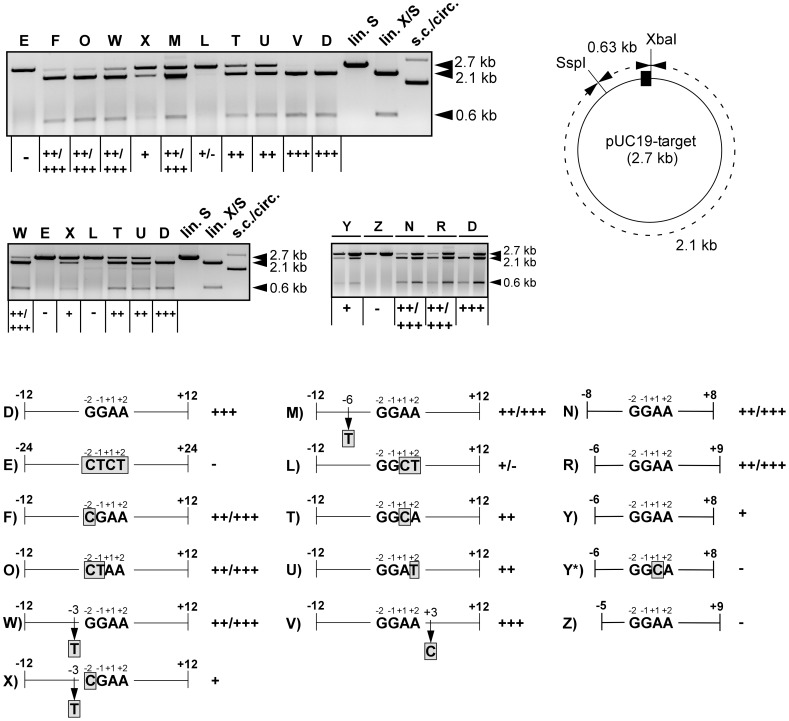
Analysis of the I-*Uma*I target site specificity. CF from induced pAP2 cells was incubated with various substrate plasmids under standard conditions for analysis of cleavage efficiencies (see Materials and Methods). Letters denote the individual substrate plasmids (*e.g.* D for pUC19-D). Reaction products were additionally cleaved with *Ssp*I to yield 2.1 and 0.63 kb fragments in case of cleavage (schematic on the right: the I-*Uma*I target site fragment is indicated as black box. *Xba*I cleaves at the right border and in combination with *Ssp*I produces fragments similar in size to those produced by I-*Uma*I/*Ssp*I). Marker lanes: lin. S, pUC19-D cleaved with *Ssp*I; lin. X/S, pUC19-D cleaved with *Xba*I/*Ssp*I; s.c./circ., uncleaved pUC19-D showing the supercoiled and circular forms. The + and - symbols refer to the cleavage efficiencies. +++, complete cleavage; ++/+++, >50% cleavage; ++, ∼50% cleavage; +, <50% cleavage; +/−, faint cleavage bands were detected; -, no detectable cleavage. The double lanes in the lower panel on the right refer to two different substrate concentrations used: for the right lane, the concentration was 3.3-fold higher than normally used. Note the inefficient cleavage of pUC19-Y. The schematic (bottom) shows the lengths and substitutions (boxed, shaded gray) of the tested constructs. Numbers refer to the positions within the target site as indicated (see Table 1).

Based on the DNA sequences bordering the observed cleavage site, we generated a series of potential target site fragments to delineate the substrate specificity of I-*Uma*I (Table S3). For this purpose, the enzyme assay was modified in that reaction products were subsequently cleaved within the plasmid backbone (see schematic in Figure 3). First, we determined the minimum target site length. This showed that sequences flanking the central-four base pairs 5′-GGAA-3′ could be shortened to 6 bp on either side (substrate plasmid pUC19-N) without strongly affecting cleavage efficiency, whereas further truncation to 5 bp abolished cleavage (pUC19-I; Table 1). However, 4 or 5 bp fragments on the left side still provided for cleavage in the presence of a 7 bp fragment on the right side (pUC19-P,R; Figure 3), but not the other way around (pUC19-Q) (Table 1). Taken together, this showed that a 15 bp sequence was sufficient for cleavage and implicated that 4 bp on the left and 6 bp on the right of the central cleavage site define the minimum target site. This was explicitly verified, although the corresponding substrate (pUC19-Y) was clearly less efficiently cleaved than the one with 7 bp on the right side (pUC19-R), indicating that position +9 contributed to the target site specificity (Figure 3). Further shortening of the left side (pUC19-Z) ultimately confirmed the minimum target site length of 14 bp *in vitro* (Figure 3). To assess further target site requirements, plasmid pUC19-M, in which position -6 was substituted within a longer region, was constructed. This showed that cleavage was only weakly affected providing evidence for contacts beyond position -6 on the left side (Figure 3). In favor of contact points beyond the 14-base pairs target site motif, a substitution of position +1 abolished cleavage within pUC19-Y (pUC19-Y*), while the same substitution in pUC19-T only partially reduced cleavage (Figure 3).

**Table 1:** Survey of tested target sites.

Identity: pUC19-	Length^1^	Sequence^2^	Cleavage efficiency^3^
A	-12/+24	CAGTTAGACG**GGAA**GACCCTATGCAGCTTTACTGTA	+++
B	−24/+24	GCGGTTTACCTTCAGTTAGACG**GGAA**GACCCTATGCAGCTTTACTGTA	+++
C	−24/+12	GCGGTTTACCTTCAGTTAGACG**GGAA**GACCCTATGC	+++
D	−12/+12	CAGTTAGACG**GGAA**GACCCTATGC	+++
E	−24/+24	GCGGTTTACCTTCAGTTAGACG**CTCT**GACCCTATGCAGCTTTACTGTA	–
F	−12/+12	CAGTTAGACG**CGAA**GACCCTATGC	++/+++
G	−11/+11	AGTTAGACG**GGAA**GACCCTATG	++/+++
H	−9/+9	TTAGACG**GGAA**GACCCTA	++/+++
I	−7/+7	AGACG**GGAA**GACCC	+/−
K	−12/+12	CAGTTAGACG**AGAA**GACCCTATGC	++/+++
L	−12/+12	CAGTTAGACG**GGCT**GACCCTATGC	+/−
M	−12/+12	CAGTTA**T**ACG**GGAA**GACCCTATGC	++/+++
N	−8/+8	TAGACG**GGAA**GACCCT	++/+++
O	−12/+12	CAGTTAGACG**CTAA**GACCCTATGC	++/+++
P	−7/+9	AGACG**GGAA**GACCCTA	++/+++
Q	−9/+7	TTAGACG**GGAA**GACCC	+/−
R	−6/+9	GACG**GGAA**GACCCTA	++/+++
T	−12/+12	CAGTTAGACG**GGCA**GACCCTATGC	++
U	−12/+12	CAGTTAGACG**GGAT**GACCCTATGC	++
V	−12/+12	CAGTTAGACG**GGAAC**ACCCTATGC	+++
W	−12/+12	CAGTTAGAC**TGGAA**GACCCTATGC	++/+++
X	−12/+12	CAGTTAGAC**TCGAA**GACCCTATGC	+
Y	−6/+8	GACG**GGAA**GACCCT	+
Y*	−6/+8	GACG**GGCA**GACCCT	–
Z	−5/+9	ACG**GGAA**GACCCTA	–

1 The numbering refers to the central-four base pairs GG_−1_A_+1_A.

2 The central-four nucleotides (or mutated derivatives) of the cleavage site are in bold face type. Nucleotide substitutions are underlined and in bold face type.

3 See legend of Figure 3 for the assignment of + and - symbols.

Substrate requirements were further analyzed based on substitutions within the central-four base pairs and adjacent positions. As expected, substitution of the GGAA motif abolished cleavage (pUC19-E in Figure 3). In particular, substitution of the two central adenine bases exhibited a strong effect (pUC19-L), whereas the two guanine bases on the left were not critical (pUC19-O in Figure 3). On the other side, individual substitutions of the two critical base pairs on the right only weakly affected cleavage (pUC19-T,U). Likewise, individual substitutions at the adjacent position −3 (pUC19-W) or +3 (pUC19-V) largely remained without effect, whereas the substitution at −3 in combination with the substitution at −2 (pUC19-F) strongly impaired cleavage (pUC19-X in Figure 3). In conclusion, this analysis provided evidence for asymmetric recognition of the two halves of the DNA target site and has shown interdependence between adjacent base pair substitutions.

### Transcriptional Regulation of I-*Uma*I

**Figure 4:**
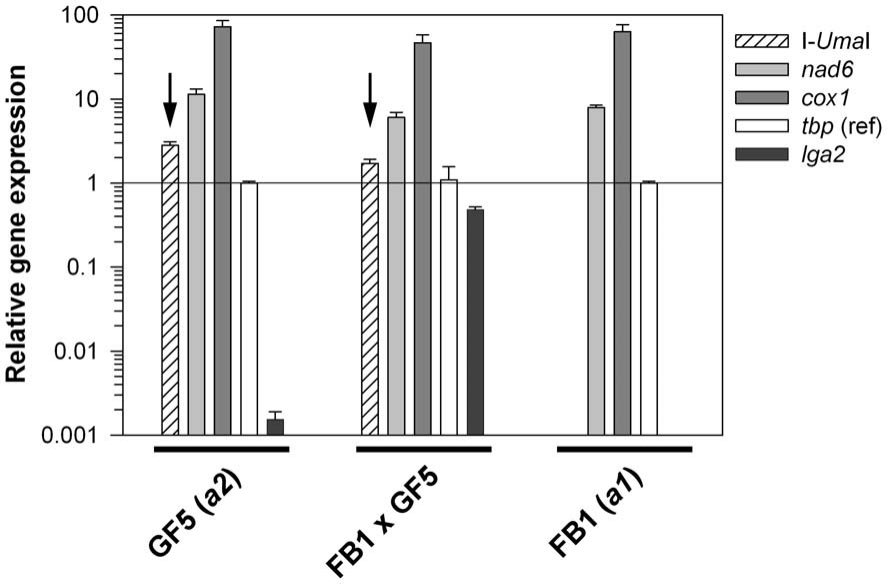
qRT-PCR analysis to assess transcription of I-*Uma*I. Comparison of transcript levels of I-*Uma*I, *nad6*, *cox1*, *tbp*, and *lga2* in dependence on sexual development. RNA was isolated from either FB1, GF5 or the mating of FB1/GF5 strains cultivated for 48 h on solid charcoal-containing complete medium. For each strain, results were normalized against the expression of the probable TATA-box binding factor gene *tbp* (*um10143*). Means and standard errors refer to four technical replicates. Note the absence of *lga2* (control for mating-dependent induction) and I-*Uma*I transcripts in strain FB1 (*a1*, F type). The detection threshold is set to 10^−3^ (relative units).

Under natural conditions, I-*Uma*I only may find a target in matings with a recipient strain that lacks the LRII1 intron. We therefore wondered whether expression of I-*Uma*I is regulated during sexual development. To test this, we applied a quantitative real-time PCR (qRT-PCR) using RNA from parental F (FB1) and W (GF5) type strains as well as from dikaryotic cells resulting from mating of these strains under standard conditions. As expected, the developmentally-regulated *a2* mating type-specific gene *lga2* was highly expressed under mating conditions, only weakly in the *a2* partner and not in the *a1* partner due to absence of the gene [21], [22]. However, the W type-associated I-*Uma*I gene was highly expressed irrespective of mating, showing a similar expression profile as two other mitochondrial genes analyzed (*cox1*, *nad6*). This indicated that I-*Uma*I is constitutively expressed and not subject of developmental regulation (Figure 4).

### Analysis of I-*Uma*II

Of further interest was the investigation of a potential HE activity encoded by the I-*Uma*II gene, which is positioned within the LRI1 intron (see Figure 1A). Like I-*Uma*I, the putative I-*Uma*II protein contains two HEG domains. According to the determined codon usage (see Table S1), no sequence adaptation was necessary for expression in *E. coli*. Specifically, among the very rarely used codons, only a single TTG triplet was found, which lies immediately next to the stop codon and therefore was not considered critical. Again, I-*Uma*II remained soluble as C-terminal His-tagged fusion in the absence of a THX domain (protein AP8) as verified from immunoblot analysis (Figure S2A). For enzymatic analysis, we used a substrate plasmid (pSLMF34) comprising the putative target site region from W type mtDNA, which flanks the LRI1 insertion site (see Figure 1A). Explicitly, the substrate plasmid contained a 531 bp fragment with the predicted cleavage site at position 315. However, enzymatic analysis using either crude or affinity-purified fractions showed no cleavage activity (Figure S2B). To exclude the possibility that products remained associated with the enzyme, reaction products were amended with SDS (0.25% w/v)/EDTA (10 mM) prior to gel electrophoresis as reported previously for I-*Cre*I [23]. Despite this, no cleavage products were detected (data not shown).

### Identification of Additional I-*Uma*I Target Sites and of Putatively Functional I-*Uma*I Homologs

Based on the identification of the defined I-*Uma*I target site we investigated the recurrence of this site elsewhere in *U. maydis* mitochondrial as well as genomic DNA. For a corresponding BLASTN search, we used the minimum target site −6/+8 (contained in pUC19-Y; see Figure 3). Within the mitochondrial genome of *U. maydis* (F type strain 521) this site was confined to exon II of the LSU rDNA. Furthermore, this site was absent from the entire genomic sequence of *U. maydis*. Only one potential I-*Uma*I target site was detected in the *um11087* ORF, namely 5′-AGACGGCA_+1_AGACCCT-3′ (data not shown). This sequence corresponds to the −7/+8 target sequence with one mismatch at position -1, and therefore, it remains to be shown whether this site is cleaved (see Table 1). To analyze the occurrence of the I-*Uma*I target site within genomes of other species we applied a BLASTN search against the (nr/nt) nucleotide collection. Interestingly, this revealed that sequence motifs matching the −7/+8 target site requirement were preferentially associated with LSU rRNA genes of members of the Ustilaginomycetes and Agaricomycetes, but almost absent from ascomycetous species (Table S4).

Next, we analyzed the occurrence of I-*Uma*I homologs applying a TBLASTN search. This revealed more than 50 candidates mainly associated with mitochondrial small subunit (SSU) or LSU rRNA genes (E-values <1e-18). Consistently, these sequences were not found positive for I-*Uma*I target sites (data not shown). To sort out candidates representing putatively functional I-*Uma*I homologs, we surveyed the highest 10 scores more closely, using the criteria applied in Table 2. This revealed interrupted I-*Uma*I target sites at the predicted exon/intron junction in only the top four candidates (Figure S3 and Table 2). Strikingly, using Rfam the corresponding introns were all classified as group II introns, as previously determined for LRII1 (Table 2; [18]). In addition, all these introns were inserted into homologous positions in LSU rDNA of these species (Figure S3 and Table 2). In further support of putatively functional homologs, an amino acid sequence alignment revealed distinct regions exclusively shared with I-*Uma*I (red bars in Figure S4). Interestingly, apart from the homolog in *Sporisorium reilianum*, representing the closest known relative to *U. maydis*, the remaining three all belong to the same order, namely the Agaricales (see Discussion). However, three out of the four corresponding ORF sequences had premature stop codons and are presumably degenerate (Table 2). Hence, only the homolog of the shiitake mushroom *Lentinula edodes* (NCBI accession no. YP_006576298) appears to be preserved. In this regard, its cognate target site might differ from the one of I-*Uma*I based on three consecutive mismatches adjacent to the minimum I-*Uma*I target site (see Figure S3). In conclusion, HEGs encoding putatively functional I-*Uma*I homologs appear to be confined to group II introns associated with a few members of the Ustilaginales and Agaricales, consistent with the widespread occurrence of predicted I-*Uma*I target sites in mitochondrial LSU rRNA genes of members of the corresponding classes.

**Table 2:** Identification of I-*Uma*I homologs.

NCBI accession no.^1^/species (phylum)^2^	Similarity:E-value^1^	Predicted lengthof HE^3^	LAGLIDADGdomains^4^	Intron type/(E-value)^5^	Hostgene	I-*Uma*I target site^6^
ACL27287 (I-*Uma*I)/*Ustilago maydis* (B)	–	336 (0)	2	Group II/(1.23e-14)	LSU	yes
FQ311469/*Sporisorium reilianum* (B)	3e−101	nd (12)	2	Group II/(1.23e−14)	LSU	yes
AB697988/*Lentinula edodes* (B)	5e−63	365 (0)	2	Group II/(2.75e−12)	LSU	yes
HQ540074/*Limacella glischra* (B)	7e−56	329 (1)	2	Group II/(2.80e−12)	LSU	yes
AF087656/*Agrocybe aegerita* (B)	4e−48	319 (4)	2	Group II/(1.37e−12)	LSU	yes
FN377860/*Glomus coronatum* (G)	3e−40	309 (1)	2	no hit	LSU	no
AF404306/*Rhizophydium sp. 136* (C)	3e−29	352 (3)	2	no hit	*cox1*	no
HQ259115/*Moniliophthora roreri* (B)	4e−27	327 (0)	2	no hit	*nad5*	no
DQ364632/*Gibberella zeae* (A)	1e−25	372 (0)	2	no hit	*cox1*	no
JN007486/*Chaetomium thermophilum* (A)	2e−25	348 (0)	2	no hit	*cox1*	no
JQ015302/*Madurella mycetomatis* (A)	8e−25	366 (0)	2	no hit	*cox2*	no

1 NCBI TBLASTN 2.2.26+ search (July 2012) against the nr data base with the I-*Uma*I protein sequence as query. Shown are the top 10 hits.

2 B: Basidiomycota; G: Glomeromycota; C: Chytridiomycota; A: Ascomycota.

3 Length in amino acids. See Figure S4 for the ORF regions derived from the indicated accession numbers. In brackets: the number of premature stop codons (UAA, UAG) within the predicted ORFs. UGA has been converted to Trp according to the mitochondrial codon usage; for FN377860 and AF404306, each one nucleotide (positions 465 and 301, respectively) has been removed to provide for a continuous ORF; nd: not determined for FQ311469 due to 7 frameshift mutations within the predicted ORF region (data not shown).

4 According to Pfam; see alignment in Figure S4.

5 According to Rfam; see reference [18] for I-*Uma*I.

6 See Figure S3; for HQ540074, the minimum target sequence on the left of the intron insertion site is fully conserved, with positions 348–401 in HQ540074 corresponding to 3360–3413 in the *U. maydis* 521 mtDNA sequence. The right border could not be aligned due to the limited sequence available in HQ540074.

## Discussion

This study has demonstrated HE activity of I-*Uma*I along with the identification of the cognate target site. I-*Uma*I is a Mg^2+^-dependent HE requiring both LAGLIDADG domains for activity. The I-*Uma*I target site precisely borders the LRII1 insertion site within exon II of the LSU rRNA gene. This substantiates a role of I-*Uma*I in the mobilization of the LRII1 intron under conditions of biparental inheritance. The minimum length of the I-*Uma*I target site is 14 bp as determined *in vitro* and thus belongs to the shortest ones reported for homing endonucleases. To this end, specific homing sites with lengths of 14 bp are known from the His-Cys box endonuclease I-*Ppo*I from *Physarum polycephalum* and the monomeric archaeal LHE I-*Dmo*I [3], [5], [24], [25]. Homing endonucleases of the LAGLIDADG family are typically associated with group I self-splicing introns [3], [5], [26], but also occur in group II introns [27] and references therein, [28], [29]. Together with recent investigation [29], this study reinforces enzymatic activity of group II intron-associated LHEs.

### Cleavage Specificity of I-*Uma*I

Homing endonucleases differ from classical restriction enzymes in that longer target sites are recognized albeit with an increased tolerance, which correlates inversely to the number of specific protein-DNA contacts [3], [6], [30], [31]. Our analysis has shown that I-*Uma*I provides for cleavage of a target site with a minimum length of 14 bp, although cleavage of longer sites is clearly favored, implying that additional DNA contacts exist in flanking regions (see Table 1). This was explicitly corroborated by mutational analysis demonstrating that critical substitutions within the minimum target site were tolerated within a longer target site region. This analysis has further provided evidence for asymmetric recognition of the target site. This is exemplified by different length requirements of the two halves flanking the site of strand cleavage as well as by the finding of unequal contributions of the central-four base pairs (see Figure 3). In agreement, I-*Uma*I has two distinct LAGLIDADG domains both being required for cleavage. Unlike homodimers, monomers are not constrained to highly symmetrical DNA targets and their homing sites tend to be less palindromic [3]. In addition, monomeric LAGLIDADG enzymes are known to interact differently with the two DNA strands in their target sequences [32]–[34]. In accordance with cleavage by other homing endonucleases [3], I-*Uma*I generates a four-nucleotide 3′-overhang (5′-GGAA-3′). By contrast, the LRII1 intron lies in the sequence context 5′-GGA-intron-A-3′, which likely reflects the initial invasion in recipient DNA. The same arrangement was seen in X1 type mtDNA resulting from recombination between parental W and F types (see Figure 1A; [18]). This makes it unlikely that the LRII1 intron is directly copied into the cleavage site, but instead might be transferred along with co-conversion of homologous flanking exon sequences, as explicitly reported for intron homing [35], [36].

### Evidence for Intron Homing in *U. maydis*

Spreading of HEGs can occur with frequencies up to 100% [5], [13]–[15]. Previously, we detected that under conditions of biparental inheritance with combinations of F/W (W/F) mitotypes, the parental F type was almost completely lost in favor of the recombinant X1 type. Thus far, under conditions of biparental inheritance, only combinations with the W type strain MF18 provided for marked inheritance of the F type, while little X1 type was produced [18]. The I-*Uma*I gene of this strain carries a frameshift mutation leading to loss of the C-terminal LAGLIDADG domain. In this study, we have shown that both domains are required for cleavage at the expected intron insertion site. This clearly correlates homing efficiency of LRII1 with the functionality of the encoded HE.

Previously, we detected additional recombinant forms of the LSU rRNA gene, which suggested homing of F type-associated introns into W type mtDNA. For example, the recombinant X2 type contains the F type-derived intron LRI1 within W type mtDNA, while the introns LRI2 and LRI3 are lacking (see Figure 1A). Furthermore, the X2 type was efficiently produced in combinations with strain MF18 under conditions of biparental inheritance [18], suggesting conversion of the LRI1 intron. We therefore attempted to determine the potential activity of the LRI1-encoded HE I-*Uma*II using the expected target site region at the splice junction between exons I and II (see Figure 1A). The corresponding sequence was shown fully conserved in four independent W type strains analyzed [18]. However, despite successful expression in *E. coli*, no cleavage activity was detected under the experimental conditions applied. It is conceivable that I-*Uma*II exhibited a strongly reduced activity *in vitro*, which escaped detection. Additionally, the possibility remains that the target site has diverged. In case of the LHE I-*Ani*I it has been shown that its optimal target site deviates in two positions from its physiological target site in the host gene of *Aspergillus nidulans* [31].

### Significance of I-*Uma*I and its Propagation

Although dependence of mitochondrial intron homing on endonuclease function has been intensively studied in yeast species [3], [5], [37] and reference therein, this study has underscored the potential of intron homing in a fungal species that normally undergoes uniparental mtDNA inheritance. Consequently, if this process would be unconstrained it would lead to inactivation of all target sites, a situation that is not encountered in *U. maydis* [10], [18]. On the other side, if mtDNA inheritance would be strictly uniparental like in animals [16], HEGs might be subject of degeneration unless their products are additionally charged with beneficial functions, such as promoting splicing of host introns through a maturase activity [6], [10], [38]. The present finding of a functional copy of I-*Uma*I therefore confirms that mtDNA exchange is well-balanced between these two extremes. To this end, a dual function, which would justify the high expression level detected for I-*Uma*I irrespective of sexual development (see Figure 4), cannot be excluded for I-*Uma*I.

Based on the current database, putatively functional or former functional homologs of I-*Uma*I are confined to members of the Ustilaginales and Agaricales representing phylogenetic distant orders within the Basidiomycota [39]. This suggests that a common ancestor intron has been acquired independently, in line with reported evidence for horizontal spread of the ω-associated HEG in Saccharomycete yeasts and the current view of horizontal transfer of mobile introns between distantly related species [5], [40]. In this regard, it is not excluded that I-*Uma*I-associated introns are more spread than anticipated from available genomic data, as suggested from the wide occurrence of predicted I-*Uma*I target sites in LSU rRNA genes of members of the Ustilaginomycetes and Agaricomycetes (see Table S4) as well as from the identification of *U. maydis* strains, which do not carry LRII1 (see Figure 1A; [18]). Therefore, it may be worthwhile to await additional genomic data to trace the history of invasion and transmission of this mobile element.

In this context, it will be exciting to examine whether maintenance of I-*Uma*I target sites was due to uniparental mtDNA inheritance or simply reflects the absence of I-*Uma*I homologs in populations of corresponding species. The finding that I-*Uma*I and homologous genes are subject of degeneration (see Table 2) implies that underlying intron homing is highly dynamic giving rise to fixation of cognate target sites [10], [40]. To this end, the only intact putatively functional I-*Uma*I homolog is from *L. edodes*. Its deduced target site is overlapping with that of I-*Uma*I such that the minimum target site is preserved, while adjacent positions on the left side differ (see Figure S3). This supports our assumption that base pair positions extending the I-*Uma*I minimum target site contribute to the cleavage specificity. To test this, further work elucidating the target site specificity of the putative *L. edodes* enzyme will be necessary. In this context, it might be particularly interesting to identify contacting amino acids within the presumed LAGLIDADG domains of the *U. maydis* and *L. edodes* proteins (see Figure S4). This may yield insight into mechanisms underlying the acquisition of new target sites.

LHEs are generally regarded as tools for genomic engineering based on their exceptional cleavage specificity. For example, they serve to introduce strand breaks to promote homologous recombination for the purpose of targeted gene disruption. In this matter, monomeric enzymes like I-*Uma*I might be more favorable for heterologous expression than their homodimeric cousins considering the smaller sizes and the fact that they do not rely upon dimerization [6].

### Conclusion

This study has provided for the functional characterization of a novel group II-associated homing endonuclease. Together with the characterization of a naturally occurring mutant variant it strongly supports the concept of rigorous intron homing under conditions of biparental inheritance. This in turn emphasizes the need for underlying control by uniparental mtDNA inheritance. Bioinformatic analyses suggest that I-*Uma*I represents a descendant of an archetype HEG that has invaded two phylogenetically distant orders consistent with the wide occurrence of predicted cognate target sites in members of the respective classes. Based on its relatively short target site length, I-*Uma*I provides a potentially valuable framework for developing variant enzymes with altered target specificities.

## Materials and Methods

### Strains, Growth Conditions and Chemicals

Growth conditions of the *U. maydis* wild-type strains 521 (*a1b1*), FB1 (*a1b1*), GF5 (*a2b13*), MF18 (*a1b17*) and mating performance were as described [18]. *E. coli* K12 strain TOP10 (Life Technologies, Karlsruhe, Germany) was used as host for plasmid amplifications and enzyme expressions. *E. coli* strains were cultivated in dYT/Ap (ampicillin; 100 µg/ml). If not further specified, all chemicals were of analytical grade and obtained from Sigma (Taufkirchen, Germany) or Roth (Karlsruhe, Germany).

### DNA and RNA Procedures

Isolation of *U. maydis* DNA and nucleic-acid procedures were done as described [41]. RNA was isolated from *U. maydis* cultivated for 2 days on solid CM charcoal plates using the TRIzol reagent (Life Technologies). Plasmids were isolated from *E. coli* using the lysis by boiling method [42]. Restriction enzymes were from NEB (New England Biolabs, Frankfurt a.M., Germany), oligonucleotides from MWG (Ebersberg, Germany). The correctness of all plasmid constructs was verified by sequencing (Sequencing Service, Faculty of Biology, LMU Munich: http://www.gi.bio.lmu.de/sequencing/).

### Expression Constructs

For pAP1, the I-*Uma*I ORF (NCBI accession no. EU921805; positions 2892–3899) was amplified (Phusion High-Fidelity DNA Polymerase, NEB) from genomic DNA of strain GF5 for cloning into pBAD102/D-TOPO (Life Technologies), using the primer combination 5′-CACCATGGATACAACTTATGATTCTAC-3′ (LRIIf2; *Nco*I site underlined)/5′-TTTACGATAACGATTCATCGTCG-3′. pAP2 was derived from pAP1 by removing the internal 377 bp *Nco*I fragment, which encodes the N-terminal thioredoxin (THX) domain. pAP1DK and pAP2DK were generated from pAP1 and pAP2, respectively, by cleavage with *Xba*I/*Nhe*I and subsequent religation of the larger 4986 bp fragment. pAP10 carries the mutant I-*Uma*I ORF of strain MF18 (NCBI accession no. EU921803; region 2884–3866) up to the native stop codon (U_610_AG). To generate pAP10, a PCR-based mutagenesis approach was applied using genomic DNA of strain MF18 to yield an ORF fragment carrying the TGA_534_ to TGG_534_ and ATA_549_ to ATG_549_ substitutions within the frame-shifted 3′ region. Overlapping primers 5′- GTAGTTTAATAACATCACGAAGATCATCCAGGTAATTCCACC-3′ (reverse) and 5′-GGAATTACCTGGATGATCTTCGTGATGTTATTAAACTACACCG-3′ (forward), comprising the two substitutions (underlined), were used in combination with outer primers LRIIf2 (see above) and 5′-GCCGAAATTGAAATGATCCTTC-3′ (reverse), respectively. Gel-purified fragments were annealed prior to a second PCR using the outer primers. The resulting PCR product was ligated into pBAD102/D-TOPO and the internal 377 bp *Nco*I fragment was removed subsequently. Due to an internal deletion within the region of the inner primers, the same experimental approach was applied, using the generated plasmid as template, with the outer reverse primer 5′- GATGACCGGTACGCGTAGAATCG-3′, which is antiparallel to the 780–802 region in pBAD102/D-TOPO. The PCR product was cleaved with *Bgl*II/*Hind*III to correct the corresponding region in pAP10.

### Substrate Plasmids

A 1075 bp DNA fragment comprising the predicted I-*Uma*I target site was amplified from genomic DNA of *U. maydis* strain 521 (F type) using the primer combination 5′-GGAATTCCATATGCTCCTCGCCGAATACGAGAGG-3′)/5′-GGAATTCCATATGTCCCAGTCAAACTGACCACC-3′ (*Nde*I sites underlined) and inserted into the *Nde*I site of pSL1180 (GE Healthcare, Darmstadt, Germany) to yield pSL521. For the collection of short I-*Uma*I target site plasmids, termed pUC19-A to Z, matching oligonucleotides (3.4 µM each; see Table S3) were combined in restriction enzyme buffer containing bovine serum albumine (BSA; 1 mg/ml) and protruding ends were filled in the presence of Klenow enzyme (NEB). After heat inactivation (10 min, 75°C), double-stranded DNA was restricted with *Bam*HI/*Xba*I, followed by heat inactivation (20 min, 85°C) and purification on a matrix (JetSorb; GENOMED, Löhne, Germany). Cleaved fragments were ligated in the presence of T4 ligase (NEB) into the compatible sites of pUC19 (GE Healthcare).

### Enzyme Preparations

Overnight cultures (200 rpm, 37°C) of the various *E. coli* strains were transferred to 50 ml fresh dYT/Ap medium at starting optical densities at 600 nm (OD_600_) of 0.1 and incubated to densities of 0.5. Cultures were subsequently supplemented with or without 0.01% (w/v) arabinose (Ara) and incubated for additional 90 min. For control, the non-transformed *E. coli* TOP10 strain treated with 0.01% (w/v) Ara in dYT was used. Cells were collected by centrifugation (1700 g; 4°C), washed in 10 ml ice-cold extraction buffer (EB: 50 mM Tris-HCl, pH 8.0; 50 mM NaCl; 4% glycerol; v/v), resuspended in 3 ml ice-cold EB+ (EB supplemented with 0.1 mM EDTA, 2 mM DTT, 1x Complete Protease Inhibitor; Roche, Mannheim, Germany) and stored frozen at -80°C. For enzyme extraction, pellets were subjected to four cycles of freeze-thawing, followed by sonification, using a Bandelin Sonopulse HD3080 sonifier (running at 50%, 5 sec/10 sec pulse/pause intervals for 10 min on ice). Homogenates were centrifuged (15 min, 8500 g; 4°C) and supernatants of the crude fraction (CF) were stored frozen (−80°C) in aliquots. For affinity purification, 550 µl of CF were applied to Ni-NTA spin columns (Qiagen, Hildesheim, Germany) as specified by the manufacturer. Columns were equilibrated in EB+ supplemented with 8 mM imidazole prior to application of the CF samples. Columns were washed with three volumes (500 µl) of EB/20 mM imidazole, followed by elution with two volumes (230 µl) of EB/250 mM imidazole supplemented with 1x Complete Protease Inhibitor to yield the purified fraction (PF). The combined eluates were used for enzyme analysis. Protein concentrations were determined using the Bio-Rad Protein Assay (Bio-Rad, Munich, Germany) with BSA as standard. Protein concentrations of the enzyme fractions were 1749+/−58 µg/ml for CF and 27.2+/−5.3 µg/ml for PF.

### Enzyme Assay

The applied enzymatic conditions basically followed the protocol of Monteilhet *et al.* [37]. If not further specified, enzyme preparations (10 to 20 µl) were incubated in the presence of substrate plasmid (50–100 ng) in 60 to 100 µl reaction buffer (EB supplemented with 0.1 mg/ml BSA, 10 mM MgCl_2_) for 30 min at 30°C. Reaction mixtures were either proceeded by phenol/chloroform extraction, followed by ethanol precipitation, or by exposure to 85°C (5 min), followed by purification of plasmid-containing supernatants on a matrix (JetSorb; GENOMED). Both treatments gave the same product yields (data not shown). In either case, DNA was dissolved in 0.5x TE (Tris-EDTA)/RNase (10 µg/ml) and applied to agarose (1%)/ethidium bromide gel electrophoresis. Alternatively, reaction products were digested with *Ssp*I prior to gel documentation. Substrate plasmid pUC19-D either non-digested or cleaved with either *Ssp*I or *Ssp*I/*Xba*I, and pSL521 either non-digested or cleaved with *Bam*HI were used for controls. Size markers were phage λ DNA digested with *Pst*I (0.25 µg) or GeneRuler™ DNA Ladder Mix (1 µg; Fermentas, St. Leon-Rot, Germany).

### Determination of the Target Cleavage Site

Linearized target plasmid pSL521 (ca. 1 µg) received from digestion with AP1 and AP2, respectively, was extracted from agarose gels and treated with T4 DNA polymerase (0.5 units; NEB) for 10 min at 4°C in the absence of dNTPs, followed by 30 min incubation at 12°C in the presence of 0.2 mM dNTPs. Blunt-ended plasmids were religated either in the absence or presence of the 692 bp *Dra*I fragment isolated from pUC19. The cleavage sites of three independent clones (two linearized by AP1 and AP2, respectively, and ligated to the *Dra*I-fragment, and one linearized by AP2 and directly religated) were analyzed by sequencing.

### Quantitative Real-time PCR (qRT-PCR) Analysis

Total RNA (2 µg) was treated with DNase (Ambion, Life Technologies). qRT-PCR analysis was applied using the SuperScript III First-Strand Synthesis SuperMix (Life Technologies) in the presence of hexamer primers and the Platinum SYBR qPCR Supermix-UDG kit (Life Technologies) in the presence of 10 nM fluorescein (Bio-Rad, Munich, Germany). Gene specific primers for I-*Uma*I were: 5′-GGAATTACCTGAATGATCTTCGTGA-3′/5′-CCGAAATTGAAATGATCCTTCTCCA-3′. Specific primers against *nad6, cox1, lga2* and *tbp* have been described [43].

### Protein Gels and Immunodetection

Protein amounts as specified in the text were applied to SDS-PAGE (10%) for immunodetection as described [41]. A monoclonal anti-His(C-term) antibody (Life Technologies; 1∶5000) and a goat anti-mouse immunoglobulin G-horseradish peroxidase HRP conjugate (1∶10000; Promega, Mannheim, Germany) were used as primary and secondary antibodies, respectively. Protein markers peqGOLD IV or V (Peqlab, Erlangen, Germany) were used as size markers.

### Databases

Protein and nucleotide sequences were compared using the NCBI BLAST database (http://blast.ncbi.nlm.nih.gov/Blast.cgi). Mitochondrial sequences of *U. maydis* were retrieved from NCBI under accession no. DQ157700 if not further specified in the text. Protein subdomains were predicted by Pfam (http://pfam.sanger.ac.uk/search). Analysis of intronic regions was done with the software provided by Rfam (http://rfam.sanger.ac.uk/search). ClustalX was used for comparative sequence alignments (http://www.clustal.org/). The BROAD database (http://www.broadinstitute.org/annotation/genome/ustilago_maydis/Home.html) was used to search *U. maydis* genomic sequences for I-*Uma*I sites. *U. maydis* gene sequences (um numbers) were retrieved from the MIPS (Munich Information Center for Protein Sequences) *Ustilago maydis* Genome Database (MUMDB; http://mips.helmholtz-muenchen.de/genre/proj/ustilago).

## Supporting Information

Figure S1 **Cleavage conditions for I-** ***Uma*** **I.** (A) Test of different temperature conditions. CF from induced pAP2 cells was incubated with pUC19-B (see Table 1) under standard conditions at either 30°C or 37°C using different amounts of CF (18, 9, 3.6 µg from left to right). Marker lanes: s.c./circ., uncleaved pUC19-B showing the supercoiled and circular forms; lin. X/S, pUC19-B cleaved with *Xba*I/*Ssp*I; lin. S, pUC19-B cleaved with *Ssp*I. Panel on the right: test of different pH conditions. Replicate samples for pH 8.0. (B) Cleavage of different substrate plasmids (see Table 1) either supercoiled (1–4) or precleaved with *Ssp*I (5–8). Marker lanes: lin. S, pUC19-D cleaved with *Ssp*I; lin. X/S, pUC19-D cleaved with *Xba*I/*Ssp*I. Note that the cleavage efficiencies of the different substrate plasmids were maintained under the two conditions. (C) Metal ion requirement for I-*Uma*I. CF from induced pAP2 cells was incubated with substrate plasmid pUC19-D in the presence of different cations under standard conditions. MgCl_2_, MnCl_2_, ZnCl_2_, CoCl_2_, CaCl_2_, CuCl_2_: each 10 mM (1.3 mM for CoCl_2_). Panel on the right: test of different MgCl_2_ concentrations: lanes 1–6∶0, 0.5, 1, 2, 5, 10 mM. (A–C) Reaction products (1–4 in part B) were cleaved with *Ssp*I resulting in 2.1 and 0.63 kb fragments in case of cleavage by I-*Uma*I. All lanes per image are from the same gel. The efficiency of the cleavage is seen from the disappearance of the 2.7 kb band in favor of the 2.1 and 0.63 kb bands (see schematic in Figure 3).
(TIF)

Figure S2 **Enzymatic analysis of I-** ***Uma*** **II.** (A) Verification of I-*Uma*II expression. CF (1,2) or PF (3) from either non-induced (−) or induced (+) pAP8 cells were applied to SDS-PAGE for subsequent immunoblot analysis to detect His-tagged I-*Uma*II. CF from non-transformed *E. coli* cells incubated under inducing conditions served as a negative control (4). Approximately 18 µg protein were loaded for CF and 0.25 µg for PF. The upper band corresponds to the predicted molecular mass of AP8 (34.7 kDa). (B) Enzyme assay with CF (1, 2) and PF (4) from either non-induced (−) or induced (+) pAP8 cells. CF from non-transformed *E. coli* cells incubated under inducing conditions served as a negative control (3). The used substrate plasmid pSLMF34 (3959 bp) uncut (K1) or linearized (K2) served as marker. See Figure 2A for the used DNA ladder. All lanes are from the same gel.
(TIF)

Figure S3 **Insertion sites of introns containing predicted I-** ***Uma*** **I homologs.** Shown are the insertion sites of intronic regions containing the predicted I-*Uma*I homologs in mtDNA of *S. reilianum*, *L. edodes*, and *A. aegerita* (NCBI accession numbers are written nearby). The I-*Uma*I target site (−6/+9; see Table 1) is typed in bold face and shaded gray. The central-four bases are typed in red. Numbers refer to positions in the corresponding NCBI sequences. Intronic regions are depicted by black horizontal lines, with the thick blue arrows marking the positions of the corresponding HEGs (drawn to scale; start and end positions are indicated). Dots mark identical bases in the sequence alignments, with the upper sequence corresponding to the *U. maydis* F type (NCBI accession no. DQ157700). In case of *A. aegerita*, the intron start and end positions have been shifted upstream by 2 bp relative to the sequence in AF087656 to match the intron/exon borders experimentally confirmed for *U. maydis* [18].
(TIF)

Figure S4 **Sequence alignment of predicted I-** ***Uma*** **I homologs.** The amino acid alignment includes I-*Uma*I and predicted homologs (see Table 2). Identities of ≥40% are shaded gray. Gaps have been inserted to maximize the alignment. Amino acids exclusively conserved between I-*Uma*I and its three closest homologs shown in this alignment are typed in red. Corresponding regions (interruptions by max. one amino acid, with the conserved residue occurring in max. one additional row) are further denoted by red bars above the I-*Uma*I sequence. The sequence of *S. reilianum* has been omitted due to the accumulation of multiple frameshift mutations (see Table 2). All letters and regions marked in red also exist in the *S. reilianum* sequence except for R_105_ (referred to I-*Uma*I), E_231_, K_255_, which map to regions of predicted frameshifts. LAGLIDADG motifs are marked in blue. The alignment does not match the predicted first LAGLIDADG motifs deduced from the HQ540074 and AF087656 sequences, which by Pfam lie within amino acid positions 53–135 and 8–101, respectively. Premature stops in the predicted amino acid sequences are indicated by asterisks (see Table 2). The corresponding ORF regions from which the protein sequences were deduced are: FQ311469∶78810–79834, AB697988∶55865–56962, HQ540074∶984–1973, AF087656∶9784–10743, FN377860∶2520–3450, AF404306∶33766–34825, HQ259115∶28041–29024, DQ364632∶67072–68190, JN007486∶77114–78160, JQ015302∶16802–17902. See also Table 2 (footnote 3) for the conversion of ORF regions.
(TIF)

Table S1 **Codon usage of the mitochondrial genome of** ***U. maydis*** **.**
(DOC)

Table S2 **Differences in the codon usage between** ***U. maydis*** **and** ***S. cerevisiae*** **.**
(DOC)

Table S3 **Primers for target site analysis.**
(DOC)

Table S4 **Occurrence of potential I-** ***Uma*** **I target sites.**
(DOC)

M&M S1 **Enzyme assay under different pH conditions.**
(DOC)

M&M S2 **Construction of pAP8 and substrate plasmid pSLMF34.**
(DOC)
