# Supplementary figures and images for: The Mitochondrial LSU rRNA Group II Intron of Ustilago maydis Encodes an Active Homing Endonuclease Likely Involved in Intron Mobility
Source: PLoS One. 2012 Nov 14;7(11):e49551. doi: 10.1371/journal.pone.0049551 (PMC3498182; doi:10.1371/journal.pone.0049551)

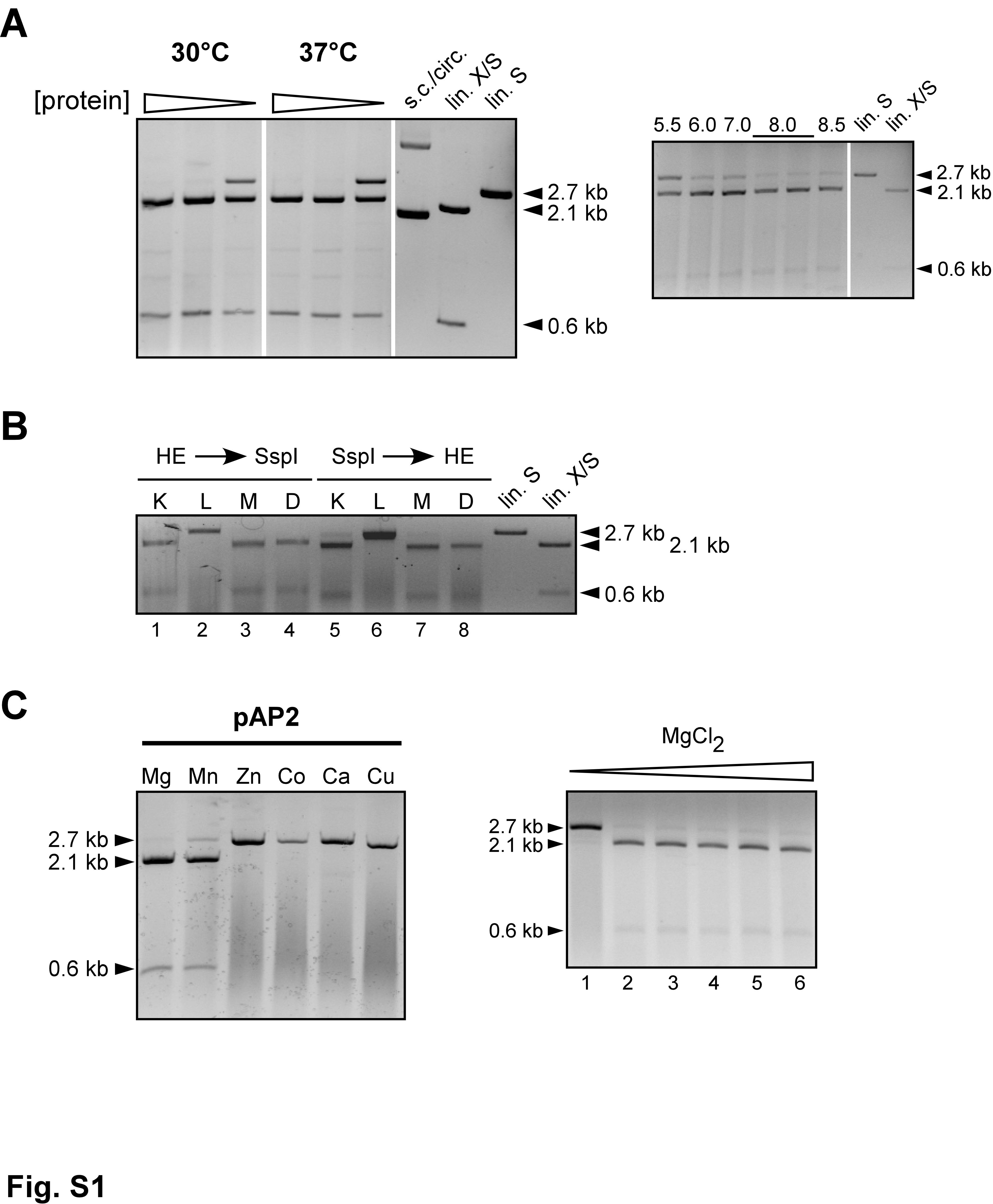

Supplement: Figure S1 — Cleavage conditions for I- Uma I. (A) Test of different temperature conditions. CF from induced pAP2 cells was incubated with pUC19-B (see Table 1) under standard conditions at either 30°C or 37°C using different amounts of CF (18, 9, 3.6 µg from left to right). Marker lanes: s.c./circ., uncleaved pUC19-B showing the supercoiled and circular forms; lin. X/S, pUC19-B cleaved with XbaI/SspI; lin. S, pUC19-B cleaved with SspI. Panel on the right: test of different pH conditions. Replicate samples for pH 8.0. (B) Cleavage of different substrate plasmids (see Table 1) either supercoiled (1–4) or precleaved with SspI (5–8). Marker lanes: lin. S, pUC19-D cleaved with SspI; lin. X/S, pUC19-D cleaved with XbaI/SspI. Note that the cleavage efficiencies of the different substrate plasmids were maintained under the two conditions. (C) Metal ion requirement for I-UmaI. CF from induced pAP2 cells was incubated with substrate plasmid pUC19-D in the presence of different cations under standard conditions. MgCl2, MnCl2, ZnCl2, CoCl2, CaCl2, CuCl2: each 10 mM (1.3 mM for CoCl2). Panel on the right: test of different MgCl2 concentrations: lanes 1–6∶0, 0.5, 1, 2, 5, 10 mM. (A–C) Reaction products (1–4 in part B) were cleaved with SspI resulting in 2.1 and 0.63 kb fragments in case of cleavage by I-UmaI. All lanes per image are from the same gel. The efficiency of the cleavage is seen from the disappearance of the 2.7 kb band in favor of the 2.1 and 0.63 kb bands (see schematic in Figure 3). (TIF) [file pone.0049551.s001.tif]

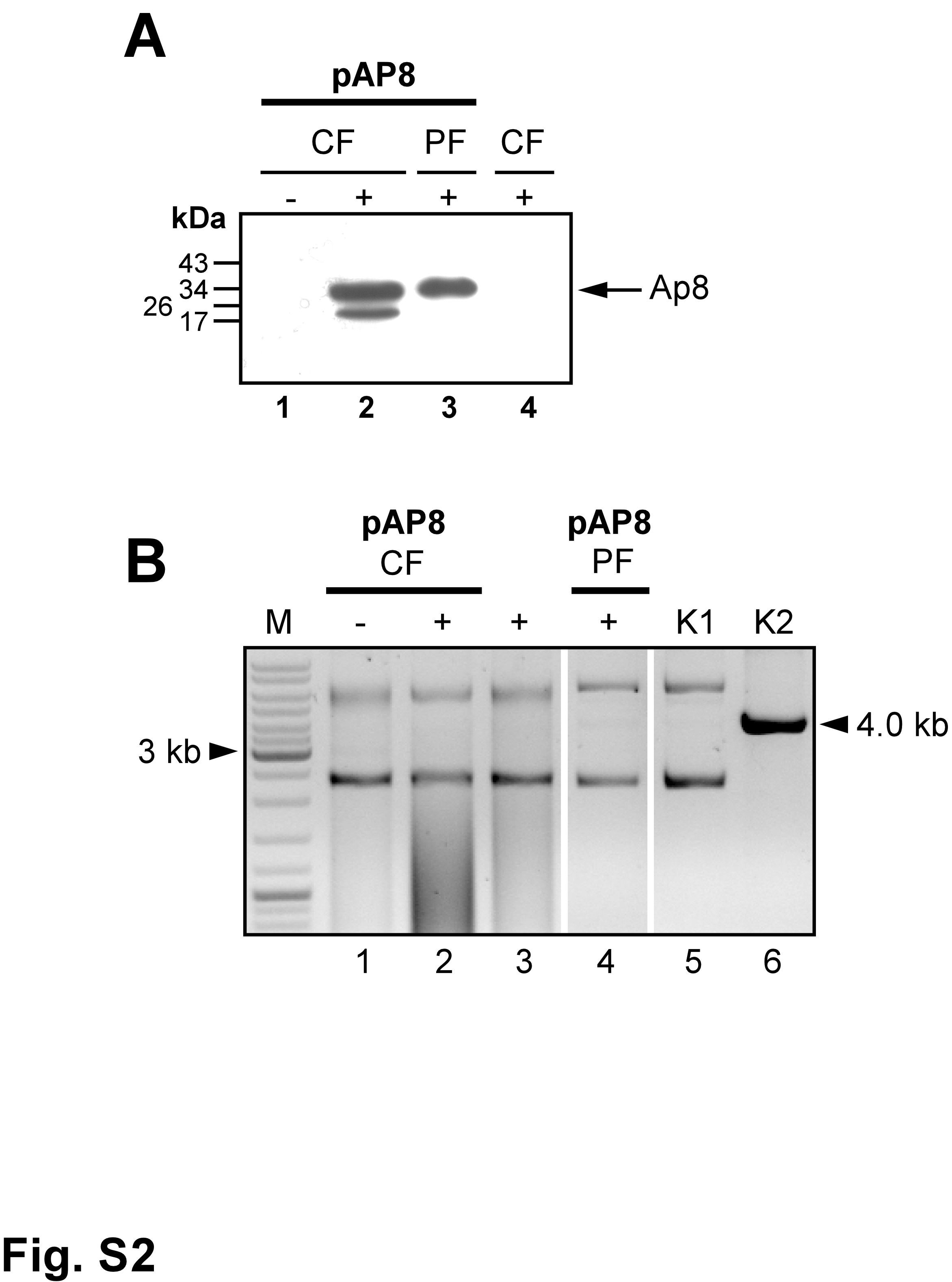

Supplement: Figure S2 — Enzymatic analysis of I- Uma II. (A) Verification of I-UmaII expression. CF (1,2) or PF (3) from either non-induced (−) or induced (+) pAP8 cells were applied to SDS-PAGE for subsequent immunoblot analysis to detect His-tagged I-UmaII. CF from non-transformed E. coli cells incubated under inducing conditions served as a negative control (4). Approximately 18 µg protein were loaded for CF and 0.25 µg for PF. The upper band corresponds to the predicted molecular mass of AP8 (34.7 kDa). (B) Enzyme assay with CF (1, 2) and PF (4) from either non-induced (−) or induced (+) pAP8 cells. CF from non-transformed E. coli cells incubated under inducing conditions served as a negative control (3). The used substrate plasmid pSLMF34 (3959 bp) uncut (K1) or linearized (K2) served as marker. See Figure 2A for the used DNA ladder. All lanes are from the same gel. (TIF) [file pone.0049551.s002.tif]

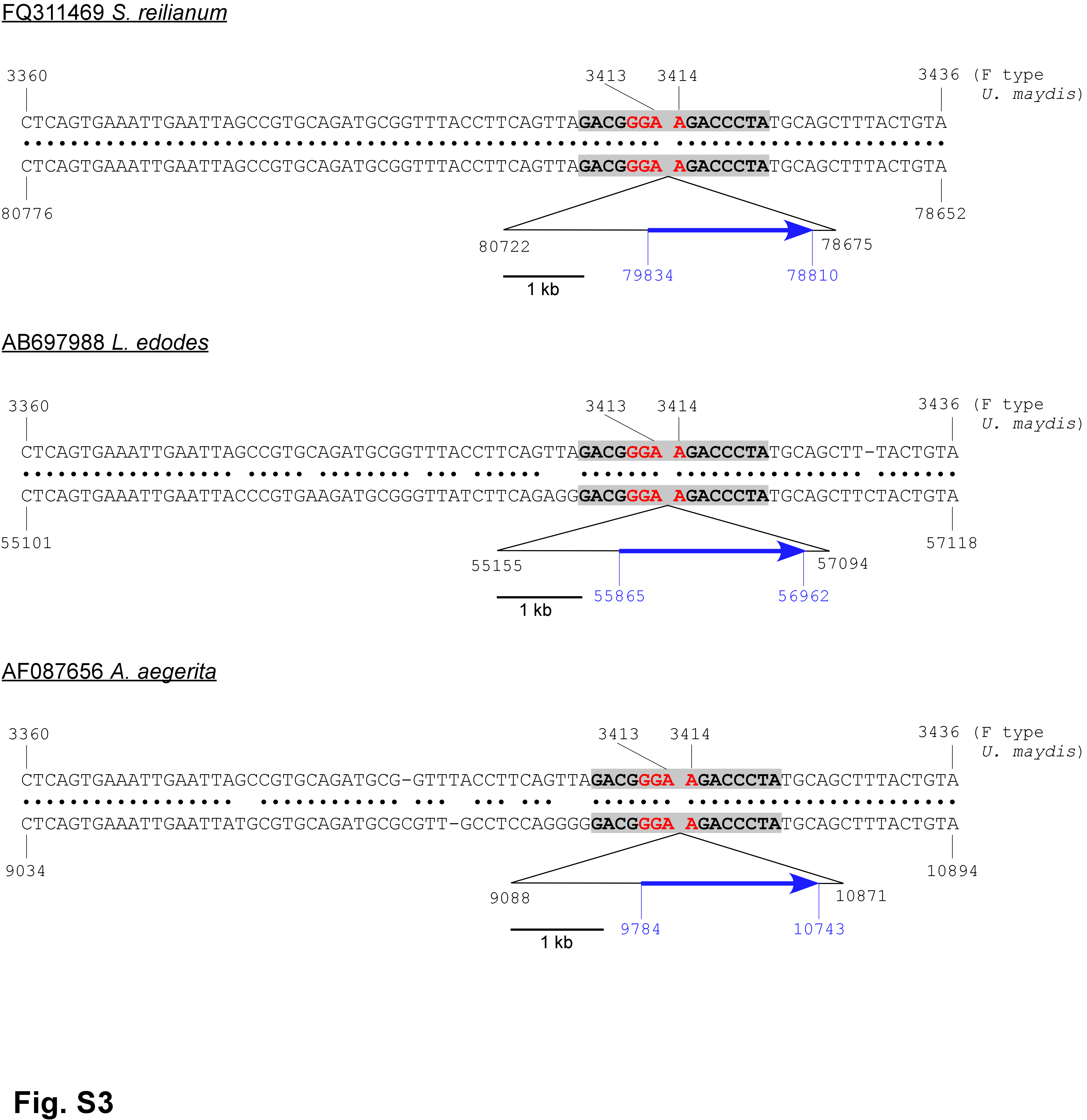

Supplement: Figure S3 — Insertion sites of introns containing predicted I- Uma I homologs. Shown are the insertion sites of intronic regions containing the predicted I-UmaI homologs in mtDNA of S. reilianum, L. edodes, and A. aegerita (NCBI accession numbers are written nearby). The I-UmaI target site (−6/+9; see Table 1) is typed in bold face and shaded gray. The central-four bases are typed in red. Numbers refer to positions in the corresponding NCBI sequences. Intronic regions are depicted by black horizontal lines, with the thick blue arrows marking the positions of the corresponding HEGs (drawn to scale; start and end positions are indicated). Dots mark identical bases in the sequence alignments, with the upper sequence corresponding to the U. maydis F type (NCBI accession no. DQ157700). In case of A. aegerita, the intron start and end positions have been shifted upstream by 2 bp relative to the sequence in AF087656 to match the intron/exon borders experimentally confirmed for U. maydis [18]. (TIF) [file pone.0049551.s003.tif]

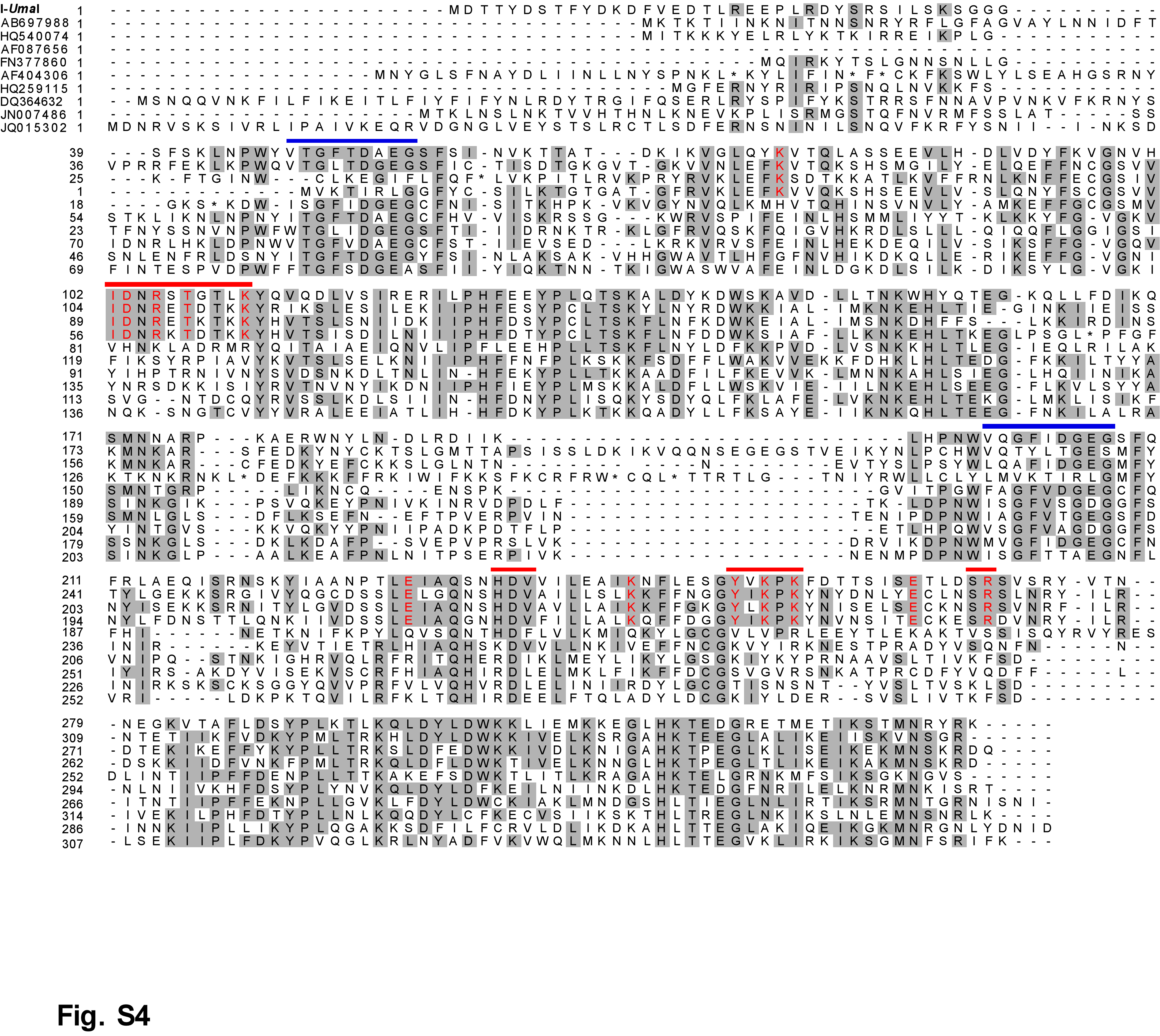

Supplement: Figure S4 — Sequence alignment of predicted I- Uma I homologs. The amino acid alignment includes I-UmaI and predicted homologs (see Table 2). Identities of ≥40% are shaded gray. Gaps have been inserted to maximize the alignment. Amino acids exclusively conserved between I-UmaI and its three closest homologs shown in this alignment are typed in red. Corresponding regions (interruptions by max. one amino acid, with the conserved residue occurring in max. one additional row) are further denoted by red bars above the I-UmaI sequence. The sequence of S. reilianum has been omitted due to the accumulation of multiple frameshift mutations (see Table 2). All letters and regions marked in red also exist in the S. reilianum sequence except for R105 (referred to I-UmaI), E231, K255, which map to regions of predicted frameshifts. LAGLIDADG motifs are marked in blue. The alignment does not match the predicted first LAGLIDADG motifs deduced from the HQ540074 and AF087656 sequences, which by Pfam lie within amino acid positions 53–135 and 8–101, respectively. Premature stops in the predicted amino acid sequences are indicated by asterisks (see Table 2). The corresponding ORF regions from which the protein sequences were deduced are: FQ311469∶78810–79834, AB697988∶55865–56962, HQ540074∶984–1973, AF087656∶9784–10743, FN377860∶2520–3450, AF404306∶33766–34825, HQ259115∶28041–29024, DQ364632∶67072–68190, JN007486∶77114–78160, JQ015302∶16802–17902. See also Table 2 (footnote 3) for the conversion of ORF regions. (TIF) [file pone.0049551.s004.tif]
