# Supplementary material for: The Mitochondrial LSU rRNA Group II Intron of Ustilago maydis Encodes an Active Homing Endonuclease Likely Involved in Intron Mobility
Source: PLoS One. 2012 Nov 14;7(11):e49551. doi: 10.1371/journal.pone.0049551 (PMC3498182; doi:10.1371/journal.pone.0049551)
Supplement: Table S1 — Codon usage of the mitochondrial genome of U. maydis . (DOC) [file pone.0049551.s005.doc]

# Table S1. Codon usage of the mitochondrial genome of *U. maydis.*

|  | **aa** | **Rps3 266** | **Nad1 331** | **Nad2 538** | **Nad3 143** | **Nad4 484** | **Nad4L 93** | **Nad5 674** | **Nad6 226** | **CoxI 528** | **CoxII 255** | **CoxIII 275** | **Atp6 254** | **Atp8 48** | **Atp9 73** | **Cob 393** | **I-*Uma*I 336** | **All 11 HEGs of strain 521** |
| --- | --- | --- | --- | --- | --- | --- | --- | --- | --- | --- | --- | --- | --- | --- | --- | --- | --- | --- |
| Codon |  |  |  |  |  |  |  |  |  |  |  |  |  |  |  |  |  |  |
| TTA | Leu | - | - | 1 | 1 | - | - | 1 | - | - | - | - | - | - | - | - | - | 2 |
| TTG | Leu | - | - | - | - | - | - | - | - | - | - | - | - | - | - | - | - | 4 |
| CTT | Leu | 3 | 10 | 22 | 5 | 6 | 5 | 21 | 16 | 7 | 5 | 4 | 9 | - | 3 | 11 | 7 | 87 |
| CTC | Leu | 1 | - | - | - | 2 | - | - | 1 | - | - | 1 | - | - | - | - | - | 14 |
| CTA | Leu | 16 | 41 | 64 | 12 | 73 | 13 | 89 | 19 | 53 | 17 | 30 | 33 | 9 | 10 | 41 | 19 | 177 |
| CTG | Leu | 2 | 2 | 4 | - | - | - | 1 | 3 | 3 | - | - | 1 | - | 0 | 3 | 6 | 9 |
| ACT | Thr | 17 | 2 | 16 | 2 | 8 | - | 13 | 9 | 5 | 3 | 6 | 5 | 1 | 2 | 6 | 5 | 95 |
| ACC | Thr | - | - | 1 | - | 1 | - | 2 | - | - | - | - | - | - | - | - | - | 7 |
| ACA | Thr | 10 | 12 | 25 | 8 | 20 | 2 | 29 | 6 | 26 | 9 | 17 | 9 | - | - | 12 | 19 | 71 |
| ACG | Thr | 3 | - | 1 | 1 | - | - | 1 | - | - | - | - | 1 | - | - | - | 2 | 6 |
| CGA | Arg | 4 | 5 | 7 | 3 | 11 | 4 | 9 | - | 7 | 2 | 5 | 3 | 1 | 2 | 12 | 4 | 55 |
| CGC | Arg | - | - | - | - | - | - | 1 | - | - | - | - | - | - | - | - | 1 | 6 |
| CGT | Arg | 2 | 3 | - | - | 4 | - | 2 | 1 | 1 | 1 | - | - | - | - | 1 | 9 | 41 |
| CGG | Arg | - | - | - | - | - | - | - | - | 2 | 2 | - | 1 | - | - | - | 2 | 5 |
| AGA | Arg | - | - | - | - | - | - | - | - | - | - | - | - | - | - | - | - | - |
| AGG | Arg | - | - | - | 1 | - | - | - | - | - | - | - | - | - | - | - | - | 3 |
| ATT | Ile | 12 | 29 | 43 | 8 | 38 | 15 | 52 | 14 | 25 | 16 | 11 | 19 | 1 | - | 28 | 14 | 178 |
| ATC | Ile | 4 | 3 | 10 | 4 | 4 | - | 7 | 4 | 14 | 8 | 10 | 9 | 2 | 6 | 6 | 3 | 29 |
| ATA | Ile | - | - | 1 | - | - | - | 1 | - | - | - | - | - | - | - | - | 1 | 16 |
| TGA | Stop | - | - | - | - | - | - | - | - | - | - | - | - | - | - | - | - | - |

Table S1 refers to amino acids (aa) that are subject of non-standard codon usage in the mitochondrial genome of *S. cerevisiae*. All nucleotide sequences analyzed were derived from the mitochondrial genome sequence of *U. maydis* strain 521 according to the annotation of Kennell and Böhmer (NCBI accession no. DQ157700). The presented codon usage is based on sequence alignments of five mitochondria-encoded proteins (*U. maydis* Atp6, Cob, CoxI, CoxII, CoxIII) with their homologues sequences in *S. cerevisiae*. Numbers refer to the total number of each codon used in the corresponding ORF region. Predicted lengths in amino acids of mitochondria-encoded proteins are indicated by the numbers on top. Differences to the mitochondrial codon usage of *S. cerevisiae* are compiled in Table S2.
