# Supplementary material for: The Mitochondrial LSU rRNA Group II Intron of Ustilago maydis Encodes an Active Homing Endonuclease Likely Involved in Intron Mobility
Source: PLoS One. 2012 Nov 14;7(11):e49551. doi: 10.1371/journal.pone.0049551 (PMC3498182; doi:10.1371/journal.pone.0049551)
Supplement: Table S2 — Differences in the codon usage between U. maydis and S. cerevisiae . (DOC) [file pone.0049551.s006.doc]

**Table S2.** Differences in the codon usage between *U. maydis* and *S. cerevisiae.*

| **Codon** | **U. maydis** | **S. cerevisiae** | **Standard** |
| --- | --- | --- | --- |
| CTN/ ACN^1^ | Leu/ Thr | Thr | Leu/ Thr |
| TTA^1^ | very rare | Leu | Leu |
| CGN^2^ | Arg | not or rarely used | Arg |
| AGA^2^ | not used | Arg | Arg |
| ATA^3^ | very rare (Ile) | Met | Ile |
| TGA^4^ | not used | Trp | Stop |

^1^The amino acid Leu is predominantly encoded by CTA/T in *U. maydis*. This contrasts from *S. cerevisiae* in which CTN encodes Thr (although rarely), while TTA, being very rarely used in *U. maydis*, is the predominant codon for Leu in *S. cerevisiae* (<http://www.imb-jena.de/~sweta/genetic_code_and_evolution/mitochondrial_code.html>). In *U. maydis*, Thr is most frequently encoded by ACA/T.

^2^The amino acid Arg is predominantly encoded by CGA/T in *U. maydis*, whereas AGA is not used. In *S. cerevisiae*, CGA/C are not and CGT/G rarely used, while AGA is most frequently used for Arg.

In *U. maydis*, the preferential use of CTA for Leu is consistent with a tRNA recognizing CUA (annotated under NCBI accession no. DQ157700). The preferential use of ACA for Thr and CGA for Arg is consistent with matching tRNAs. For the very rarely occurring TTA/G and AGG codons matching tRNAs are absent. It is formally possible that tRNAs recognizing UUC (Phe) and AGC (Ser) are used. These codons are used only for the very N- (for Nad2 at position 2) or C-terminal ends of Nad3 (positions 142, 143) and Nad5 (position 674), respectively. Alternatively, these codons might serve as stop codons in the mitochondrial code of *U. maydis*. In this case, the protein sequence of Nad2 would start with Met at position 4 in the annotated protein sequence consistent with the BLASTP alignment between *U. maydis* Nad2 and the homologous sequences of *Sporisorium reilianum* (NCBI accession no. CBQ72565) and *Tilletia indica* (NCBI accession no. YP_001492838) starting both at amino acid position 4 referred to the *U. maydis* sequence.

^3^The amino acid Ile is predominantly encoded by ATT/C, while ATA is only very rarely used. The use of ATA for Ile is supported by an alignment of the *U. maydis* Nad2 sequence (see Table S1), in which the ATA codon of Ile_95_ is conserved in position with the homologues sequence of *Aspergillus niger*, in which ATA is frequently used for Ile (16 of 26 residues in Nad2). In addition, Ile_263_ of the C-terminal HE domain of I-*Uma*I (encoded by ATA) is conserved (either Ile or Leu) among the top five I-*Uma*I homologous sequences (see alignment in Figure S4).

^4^TGA is not used in *U. maydis* (see Table S1).
