# Supplementary material for: The Mitochondrial LSU rRNA Group II Intron of Ustilago maydis Encodes an Active Homing Endonuclease Likely Involved in Intron Mobility
Source: PLoS One. 2012 Nov 14;7(11):e49551. doi: 10.1371/journal.pone.0049551 (PMC3498182; doi:10.1371/journal.pone.0049551)
Supplement: Table S3 — Primers for target site analysis. (DOC) [file pone.0049551.s007.doc]

# Table S3. Primers for target site analysis.

| **Identity: pUC19-** | **Oligos (5’ to 3’ direction)**^1^ | **Target site length**^2^ |
| --- | --- | --- |
| A | gctctagaCAGTTAGACG**GGAA**GACCCTATGC/ cgggatccTACAGTAAAGCTGCATAGGGTC**TTCC**CGTCTA | -12/+24 |
| B | gctctagaGCGGTTTACCTTCAGTTAGACG**GGAA**GACCCT/ cgggatccTACAGTAAAGCTGCATAGGGTC**TTCC**CGTCTA | -24/+24 |
| C | gctctagaGCGGTTTACCTTCAGTTAGACG**GGAA**GACCCT/ cgggatccGCATAGGGTC**TTCC**CGTCTAACTG | -24/+12 |
| D | gctctagaCAGTTAGACG**GGAA**GACCCTATGC/ cgggatccGCATAGGGTC**TTCC**CGTCTAACTG | -12/+12 |
| E | gctctagaGCGGTTTACCTTCAGTTAGACG**ctct**GACCCT/ cgggatccTACAGTAAAGCTGCATAGGGTC**AGAG**CGTCTA | -24/+24 |
| F | gctctagaCAGTTAGACG**CGAA**GACCCTATGC/ cgggatccGCATAGGGTC**TTCG**CGTCTAACTG | -12/+12 |
| G | gctctagaGTTAGACG**GGAA**GACCCTAT/ cgggatccATAGGGTC**TTCC**CGTCTAAC | -11/+11 |
| H | gctctagaTTAGACG**GGAA**GACCCTA/ cgggatccTAGGGTC**TTCC**CGTCTAA | -9/+9 |
| I | gctctagaAGACG**GGAA**GACCCggatcccg/ cgggatccGGGTC**TTCC**CGTCTtctagagc | -7/+7 |
| K | gctctagaCAGTTAGACG**AGAA**GACCCTATGC/ cgggatccGCATAGGGTC**TTCT**CGTCTAACTG | -12/+12 |
| L | gctctagaCAGTTAGACG**GGCT**GACCCTATGC/ cgggatccGCATAGGGTC**AGCC**CGTCTAACTG | -12/+12 |
| M | gctctagaCAGTTA**T**ACG**GGAA**GACCCTATGC/ cgggatccGCATAGGGTC**TTCC**CGT**A**TAACTG | -12/+12 |
| N | gctctagaTAGACG**GGAA**GACCCTgg/ cgggatccAGGGTC**TTCC**CGTCTAtc | -8/+8 |
| O | gctctagaCAGTTAGACG**CTAA**GACCCTATGC/ cgggatccGCATAGGGTC**TTAG**CGTCTAACTG | -12/+12 |
| P | gctctagaAGACG**GGAA**GACCCTAgg/ cgggatccTAGGGTC**TTCC**CGTCTtc | -7/+9 |
| Q | gctctagaTTAGACG**GGAA**GACCCgg/ cgggatccGGGTC**TTCC**CGTCTAA | -9/+7 |
| R | gctctagaccGACG**GGAA**GACCCTAg/ cgggatccTAGGGTC**TTCC**CGTCggt | -6/+9 |
| T | gctctagaCAGTTAGACG**GGCA**GACCCTATGC/ cgggatccGCATAGGGTC**TGCC**CGTCTAACTG | -12/+12 |
| U | gctctagaCAGTTAGACG**GGAT**GACCCTATGC/ cgggatccGCATAGGGTC**ATCC**CGTCTAACTG | -12/+12 |
| V | gctctagaCAGTTAGACG**GGAAC**ACCCTATGC/ cgggatccGCATAGGGT**GTTCC**CGTCTAACTG | -12/+12 |
| W | gctctagaCAGTTAGAC**TGGAA**GACCCTATGC/ cgggatccGCATAGGGTC**TTCCA**GTCTAACTG | -12/+12 |
| X | gctctagaCAGTTAGAC**TCGAA**GACCCTATGC/ cgggatccGCATAGGGTC**TTCGA**GTCTAACTG | -12/+12 |
| Y | gctctagaccGACG**GGAA**GACCCTgg/ cgggatccAGGGTC**TTCC**CGTCggtc | -6/+8 |
| Y* | gctctagaccGACG**GGCA**GACCCTgg/ cgggatccAGGGTC**TGCC**CGTCggtc | -6/+8 |
| Z | gctctagaccACG**GGAA**GACCCTAgg/ cgggatccTAGGGTC**TTCC**CGTggtc | -5/+9 |

^1^Restriction sites (*Bam*HI and *Xba*I) are underlined. The central-four bases (or mutated derivatives) of the cleavage site are in bold face type. Nucleotide substitutions are underlined and in bold face type.

^2^The numbering refers to the central-four bases GG_-1_A_+1_A.
