# Supplementary material for: The Mitochondrial LSU rRNA Group II Intron of Ustilago maydis Encodes an Active Homing Endonuclease Likely Involved in Intron Mobility
Source: PLoS One. 2012 Nov 14;7(11):e49551. doi: 10.1371/journal.pone.0049551 (PMC3498182; doi:10.1371/journal.pone.0049551)
Supplement: Table S4 — Occurrence of potential I- Uma I target sites. (DOC) [file pone.0049551.s008.doc]

**Table S4**. Occurrence of potential I-*Uma*I target sites.

| **NCBI Accession** | **Description** | **Source** | **Identity**^1^ | **Phylum**^2^ |
| --- | --- | --- | --- | --- |
| JN227086 | *Ichthyophthirius multifiliis* strain G5 mitochondrion | Mitoch. | 1-18 | Ciliophora |
| GQ250146, GQ250145 | *Tulasnellaceae* **LSU rRNA*** | Mitoch. | 1-18 | Basidiomycota |
| GQ339576 | *Helicosporidium* sp. ex *Simulium jonesi* | Mitoch. | 1-18 | Chlorophyta |
| AY382800 | *Tulasnella* sp. 185 **LSU rRNA*** | Mitoch. | 1-18 | Basidiomycota |
| AY534215 | *Cantharellaceae* isolate HJA2170 **LSU rRNA*** | Mitoch. | 1-18 | Basidiomycota |
| AD001573 | *Cantharellus cibarius* **LSU rRNA*** | Mitoch. | 1-18 | Basidiomycota |
| AY323513 | *Craterellus tubaeformis* trh332 **LSU rRNA*** | Mitoch. | 1-18 | Basidiomycota |
| AF294707- 294710 | *Pseudozyma prolifica*, *aphidis*, *antarctica*, *rugulosa* sp. **LSU rRNA*** | Mitoch. | 1-18 | Basidiomycota |
| FQ859090 | *Piriformospora indica* DSM 11827 | Mitoch. | 2-18 | Basidiomycota |
| GQ250104-250155; FJ786685-786692; EU195339 | Various uncultured *Tulasnellaceae* clones **LSU rRNA*** | Mitoch. | 2-18 | Basidiomycota |
| HM196773, HM196774; GQ907052-907058; DQ834409-834412; AY634158-634160; AY382794-382814; AY192488-192525; AF518724, AY382795 | Various *Tulasnella* sp. **LSU rRNA*** | Mitoch. | 2-18 | Basidiomycota |
| AB495230-495243; AB495225-495227 | *Epulorhiza* sp. **LSU rRNA*** | Mitoch. | 2-18 | Basidiomycota |
| EF536375, DQ993184 | *Tilletia walkeri*, *indica* sp. **LSU rRNA*** | Mitoch. | 2-18 | Basidiomycota |
| DQ927303-927305; AF160864, AF396436 | *Tetrahymena pigmentosa*, *paravorax*, *malaccensis*, *pyriformis, thermophila* sp. | Mitoch. | 2-18 | Ciliophora |
| M58010-58011 | *Tetrahymena pyriformis* **LSU rRNA** | Mitoch. | 2-18 | Ciliophora |
| AF011783 | *Tetrahymena thermophila* 23S ribosomal RNA gene | Mitoch. | 2-18 | Ciliophora |
| X04822 | *Tetrahymena* mitochondrial DNA for 21S ribosomal RNA | Mitoch. | 2-18 | Ciliophora |
| AC084706, AC022828 | *Homo sapiens* chromosome | Complete sequence | 2-18 | Mammalia |
| AF518704 | *Chondrostereum purpureum* **LSU rRNA*** | Mitoch. | 2-18 | Basidiomycota |
| AF393151 | *Sistotrema eximum* **LSU rRNA*** | Mitoch. | 2-18 | Basidiomycota |
| AAU68149 | *Ascogaster argentifrons* 16S mitochondrial ribosomal RNA | Mitoch. | 2-18 | Insecta |
| AF345563 | *Epulorhiza albertensis* **LSU rRNA*** | Mitoch. | 2-18 | Basidiomycota |
| AF294711 | *Tilletiopsis washingtonensis* **LSU rRNA*** | Mitoch. | 2-18 | Basidiomycota |
| AY863212 | *Rhizopus oryzae* **LSU rRNA*** | Mitoch. | 2-18 | Glomeromycota |
| AY856080 | *Gloeotulasnella cystidiophora* **LSU rRNA*** | Mitoch. | 2-18 | Basidiomycota |
| AY293272 | *Steccherinum fimbriatum* **LSU rRNA*** | Mitoch. | 2-18 | Basidiomycota |
| AY293256 | *Hydnum albidum* **LSU rRNA*** | Mitoch. | 2-18 | Basidiomycota |
| AD001635 | *Sebacina* sp. uamh6444 **LSU rRNA*** | Mitoch. | 2-18 | Basidiomycota |
| AM286415 | *Yersinia enterocolitica* subsp. *enterocolitica* 8081 | Complete genome | 1-17 | Enterobacteriales |
| AY382813 | *Tulasnella violea strain KC851* **LSU rRNA*** | Mitoch. | 2-17 | Basidiomycota |
| AY382805 | *Tulasnella danica strain KC388* **LSU rRNA*** | Mitoch. | 2-17 | Basidiomycota |
| AY192504 | *Uncultured Tulasnella sp. 3032.3.1* **LSU rRNA*** | Mitoch. | 2-17 | Basidiomycota |
| AF110138 | *Nephroselmis olivacea mitochondrion* | Mitoch. | 2-17 | Chlorophyta |
| AY863213 | *Smittium culisetae mitochondrion* | Mitoch. | 2-17 | Glomeromycota |
| DQ097371 | *Flagelloscypha minutissima strain CBS823.88* **LSU rRNA*** | Mitoch. | 3-18 | Basidiomycota |
| AF518717 | *Phellodon tomentosus strain BG Thesis* **LSU rRNA** | Mitoch. | 3-18 | Basidiomycota |
| AF334748 | *Nia vibrissa* **LSU rRNA** | Mitoch. | 3-18 | Basidiomycota |
| U21859 | *Lactobacillus reuteri plasmid pGT232* | Complete sequence | 3-18 | Lactobacillales |
| V00699 | *Saccharomyces cerevisiae* **LSU rRNA*** | Mitoch. | 3-18 | Ascomycota |
| CP003012 | *Thielavia terrestris NRRL 8126 chromosome 4* | Complete sequence | 3-17 | Ascomycota |

^1^BLASTN search against the (nr/nt) nucleotide collection (short input search parameters) from April 2012 with the 18 bp region 5’-TTA**GACGGGAAGACCCT**A-3’ spanning the minimum I-*Uma*I target site (bold face type) as query (see Table 1). Only hits are listed with maximum two mismatches at the 5’ and one mismatch at the 3’ end (corresponds to the –7/+8 site), and, in addition, a non-interrupted target site. For the 2-17 and 3-17(18) identities, only hits from microbial species are shown. The hits for *U. maydis* mitochondrial sequences are not shown. All assignments to mitochondrial LSU rRNA genes are in bold face type. An asterisk indicates that the target site is contained within the region corresponding to *U. maydis* exon 2 (>70% identity to the region from 3250-3435 in the *U. maydis* 521 mtDNA sequence; see Fig. 1A). All assignments to the Basidiomycota exclusively refer to members of the Ustilaginomycetes or Agaricomycetes.

^2^Orders in case of bacteria.
