# Supplementary material for: The Mitochondrial LSU rRNA Group II Intron of Ustilago maydis Encodes an Active Homing Endonuclease Likely Involved in Intron Mobility
Source: PLoS One. 2012 Nov 14;7(11):e49551. doi: 10.1371/journal.pone.0049551 (PMC3498182; doi:10.1371/journal.pone.0049551)
Supplement: M&M S1 — Enzyme assay under different pH conditions. (DOC) [file pone.0049551.s009.doc]

**M&M S1. Enzyme assay under different pH conditions.**

Enzyme assays were performed under standard conditions as described in Materials and Methods. Buffers contained 0.1 M sodium acetate for pH values of 5.5 and 6.0, and 0.1 M Tris-HCl for pH values above.
