# Supplementary material for: The Mitochondrial LSU rRNA Group II Intron of Ustilago maydis Encodes an Active Homing Endonuclease Likely Involved in Intron Mobility
Source: PLoS One. 2012 Nov 14;7(11):e49551. doi: 10.1371/journal.pone.0049551 (PMC3498182; doi:10.1371/journal.pone.0049551)
Supplement: M&M S2 — Construction of pAP8 and substrate plasmid pSLMF34. (DOC) [file pone.0049551.s010.doc]

**M&M S2. Construction of pAP8 and substrate plasmid pSLMF34.**

For the construction of pAP8, the I-*Uma*II ORF (NCBI accession no. EU921800; positions 1076-1882) was amplified (Phusion High-Fidelity DNA Polymerase, NEB) from genomic DNA of strain FB1, using the primer combination 5’-CACCATGGCTAAAACACGACTATTTAATTTTAC-3’ (*Nco*I site underlined)/5’-CAACGTGTAGTCTTTTTTAGTAG-3’. The product was introduced into pBAD102/D-TOPO (Life Technologies), followed by cleavage with *Nco*I to remove the internal 377 bp fragment encoding the N-terminal THX domain. For this purpose, the forward primer contained an additional Ala codon next to the translational initiator ATG codon to provide for a *Nco*I site.

A 531 bp DNA fragment comprising the predicted *I-Uma*II target site was amplified from genomic DNA of *U. maydis* strain MF34 (W type) using the primer combination 5’-ggaattccatatgctagcgatagtggagagtaacgtg-3’/5’-ggaattccatatgcaccttatcatgaatagactgtgc-3’ (*Nde*I sites underlined) and inserted into the *Nde*I site of pSL1180 to yield pSLMF34. pSLMF34 either non-digested or cleaved with *Eco*RI were used for controls.
